# Plant–hummingbird interactions, floral abundance, and floral traits for three altitudinal gradients in the Americas

**DOI:** 10.1002/ecy.70511

**Published:** 2026-11-05

**Authors:** Tatiana Santander, Isabela G. Varassin, María A. Maglianesi, François Duchenne, Elisa Barreto, Emanuel Brenes, Tiago Machado‐de‐Souza, Francisco Tobar, Andrea Nieto, Stéphane A. Aubert, Holger Beck, Carolina Bello, Rafaela Bobato, Daniela A. S. Bôlla, Nicole Büttner, Ana Paula Caron, Jose A. Castro, Nelson Chaves‐Elizondo, María José Gavilanes, Esteban A. Guevara, Miriam Kaehler, Miguel Machnicki‐Reis, Andrés S. Marcayata‐Fajardo, Cauã G. de Menezes, Rafael de Oliveira, Ricardo A. C. de Oliveira, Cristian Poveda‐Pazmiño, Alejandro Restrepo‐González, Friederike Richter, Bryan G. Rojas, Luciele L. Romanowski, Romulo C. da Silva, Wellinton L. de Souza, Danila S. Veluza, Benjamin G. Weinstein, Rafael Wüest, Thais B. Zanata, Krystal Zuniga, Catherine H. Graham

**Affiliations:** ^1^ Aves y Conservación/BirdLife in Ecuador Quito Ecuador; ^2^ Departmento de Boânica Universidade Federal do Paraná Curitiba Brazil; ^3^ Escuela de Ciencias Exactas y Naturales, Universidad Estatal a Distancia (UNED), Vicerrectoría de Investigación San José Costa Rica; ^4^ Swiss Federal Institute for Forest, Snow and Landscape Research (WSL), Biodiversity and Conservation Biology Unit Birmensdorf Switzerland; ^5^ Station d'écologie théorique et expérimentale Moulis France; ^6^ Mater Natura – Instituto de Estudos Ambientais Curitiba Brazil; ^7^ Herbario Nacional de Ecuador (QCNE) Quito Ecuador; ^8^ Senckenberg Biodiversity and Climate Research Center (SBiK‐F) Frankfurt Germany; ^9^ Santa Lucia Cloud Forest Reserve Nanegal Ecuador; ^10^ Department of Environmental Systems Science ETH Zurich Switzerland; ^11^ Royal Botanical Gardens Kew Richmond UK; ^12^ National Institute for Amazonian Research (INPA) Manaus Brazil; ^13^ Un poco del Chocó – Reserve and Biological Station Gualea Ecuador; ^14^ Museu de História Natural Capão da Imbuia – MHNCI Curitiba Brazil; ^15^ Universidad de Costa Rica San José Costa Rica; ^16^ Universidad San Francisco de Quito Quito Ecuador; ^17^ Instituto Nacional de Biodiversidad INABIO Quito Ecuador; ^18^ Botanik, Staatliches Museum für Naturkunde Stuttgart – SMNS Stuttgart Germany; ^19^ Stiftung Naturschutz Berlin Berlin Germany; ^20^ Escuela de Biología Universidad del Azuay Cuenca Ecuador; ^21^ Laboratório de Ecologia Humana e etnobotânica, Universidade Federal de Santa Catarina Florianópolis Brazil; ^22^ Department of Wildlife Ecology and Conservation University of Florida Gainesville Florida USA; ^23^ Departamento de Botânica e Ecologia Universidade Federal de Mato Grosso Cuiabá Brazil

**Keywords:** Atlantic Forest‐Brazil, Chocó region‐Ecuador, Costa Rica, elevation gradient, floral traits, habitat disturbance, hummingbirds, nectar‐feeding, neotropics/tropical Andes, networks, plant–animal interaction, pollination

## Abstract

Plant–animal interactions result in many ecosystem services, such as pollination. In the western hemisphere, and especially the Neotropics, hummingbirds are important pollinators for thousands of plant species, yet there is much to be learned about the spatial and temporal variation in mechanisms influencing these interactions. We collected an extensive dataset from 39 sites across elevation gradients spanning 4.7–3522 m above sea level in three regions: the Pacific slope of the Talamanca Mountain Range in central‐southern Costa Rica, the Chocó‐Andean region of northwestern Pichincha province in Ecuador, and the Atlantic Forest in southeastern Brazil. Because these regions have very different biogeographic histories, comparing them allows us to identify general patterns of interactions. Data on flower abundance and plant–hummingbird interactions were gathered monthly for 1.5–2 years along 1.5‐km transects situated along an elevation gradient in each country during 2017 and 2024. Trait data for flowers were also measured. This effort resulted in 95,824 pairwise interactions between 715 plants and 79 hummingbird species, and also 873 interactions with another 12 bird species in Ecuador. These data are being used to develop a predictive framework on species interactions necessary to understand and conserve biodiversity and ecosystem services. These data also provide detailed information on plants important for hummingbird conservation which have, for example, been used to guide restoration projects. The data are available for reuse under CC BY 4.0 licensing. Please cite the data release on Zenodo and this data paper when reusing the dataset.

## CONFLICT OF INTEREST STATEMENT

The authors declare no conflicts of interest.

## Supporting information


Data S1.

## Data Availability

Data are available as Supporting Information in Data S1. Data are also available in Zenodo at https://doi.org/10.5281/zenodo.17092717.

