# Supplementary material for: Plant–hummingbird interactions, floral abundance, and floral traits for three altitudinal gradients in the Americas
Source: Ecology. 2026 Nov 5;107(11):e70511. doi: 10.1002/ecy.70511 (PMC13740649; doi:10.1002/ecy.70511)
Supplement: Supplementary file 1 [file ECY-107-e70511-s001.zip › Metadata_S1.pdf]

Data Paper published in *Ecology*, an Ecological Society of America journal

Metadata S1

Plant–hummingbird interactions, floral abundance, and floral traits for three altitudinal gradients in the Americas

Tatiana Santander, Isabela G. Varassin, María A. Maglianesi, François Duchenne, Elisa Barreto, Emanuel Brenes, Tiago Machado-de-Souza, Francisco Tobar, Andrea Nieto, Stéphane A. Aubert, Holger Beck, Carolina Bello, Rafaela Bobato, Daniela A.S. Bôlla, Nicole Büttner, Ana Paula Caron, Jose A. Castro, Nelson Chaves-Elizondo, María José Gavilanes, Esteban A. Guevara, Miriam Kaehler, Miguel Machnicki-Reis, Andrés S. Marcayata-Fajardo, Cauã G. de Menezes, Rafael de Oliveira, Ricardo A. C. de Oliveira, Cristian Poveda-Pazmiño, Alejandro Restrepo-González, Friederike Richter, Bryan G. Rojas, Luciele L. Romanowski, Romulo C. da Silva, Wellinton L. de Souza, Danila S. Veluza, Benjamin G.Weinstein, Rafael Wüest, Thais B. Zanata, Krystal Zuniga, and Catherine H. Graham

Introduction

Extremely high diversity in mountains in tropical regions has long fascinated ecologists and evolutionary biologists, and yet, identifying the underlying causes for this diversity remains an enigma (Janzen 1967, Humboldt and Bonpland 2013, Rahbek et al. 2019). Reductions in land area, atmospheric pressure, and air temperature at higher elevations impose constraints for most organisms, often resulting in high species turnover across elevational gradients and high biological diversity (Kattan et al. 2004, Sonne and Rahbek 2024). Mountain regions are also hotspots of environmental change hosting both large population centers and land-use

transformation for activities such as cattle grazing and agricultural development (Myers et al. 2000, Chakraborty 2021). Further, because mountains provide a variety of environmental conditions, evaluating diversity changes across montane gradients should provide a space-for-time substitution, yielding insights into how this diversity will be influenced by on-going environmental changes.

Most research across environmental gradients focuses on alpha and beta diversity, often considering trait and phylogenetic diversity, in addition to taxonomic diversity (e.g., Graham et al. 2009). In comparison, detailed knowledge of changes in species interactions and network structure across gradients is more limited (Pellissier et al. 2018). Network ecology aims to quantify patterns and drivers of species interactions in a given assemblage; these interactions, such as a pollinator visiting a given plant species, form the architecture of biodiversity (Bascompte and Jordano 2013). Thus, understanding how and why network structure varies across gradients provides insights into how these interactions influence community persistence.

In the Neotropics the intricate web of interactions between hummingbirds and their food plants evolved over millennia as a result of diffuse co-evolution resulting in a remarkable array of morphological forms and functions. Three-hundred and sixty-six extant hummingbird species interact with about 7,000 plant species spread across multiple angiosperm clades (Abrahamczyk and Kessler 2015, Rodríguez-Flores et al. 2019, Gill et al. 2024). Hummingbirds, which rely on the nectar produced by flowers, are important pollinators in the Western Hemisphere and particularly in tropical montane environments (Dellinger et al. 2023). Temperature, oxygen availability, air density and the abundance of flowers with long corollas all decrease with increasing elevation, resulting in species with: higher wing-loading, associated with flight at low air densities; larger tarsus, because birds perch more while foraging and shorter bills, because

flower corollas are shorter (Feinsinger et al. 1979, Stiles 2004, 2008, Colwell et al. 2023). In addition, a given assemblage of hummingbirds often have distinct roles in the community and preferentially forage on different subsets of flowers (Feinsinger et al. 1979, Stiles 2004, Colwell et al. 2023). Plant species pollinated by hummingbirds are spread across ~100 families and span diverse life forms (e.g. shrubs, herbs, trees, lianas), often bearing flowers with a typical bird pollination syndrome (Faegri and van der Pijl 1979). These flowers tend to be scentless, often red, yellow, or purple, with long corolla tubes, protruding stigmas and stamens, and copious amounts of sucrose-rich nectar (Grant and Grant 1968, Feinsinger and Colwell 1978, Pyke and Waser 1981, Ornelas et al. 2007, Abrahamczyk and Kessler 2015). Thus, given that each partner has important adaptations to the mutualism, hummingbirds and plants provide an excellent opportunity to explore patterns of network structure and to evaluate factors, such as resource niche partitioning, that influence the maintenance of tropical diversity (Feinsinger et al. 1979, Dalsgaard et al. 2021).

Macro-evolutionary and biogeographic processes generate distinct regional species pools. Therefore, systematically collected data across multiple regions should allow us to identify emergent patterns (or lack thereof) of network structure across elevational gradients to begin to understand the extent to which general principles or mechanisms influence high tropical mountain diversity. We present data from three tropical mountainous regions: the Brazilian Atlantic Forest, the Ecuadorian Andes and the Costa Rican Highlands. In these different geographic regions, hummingbirds and characteristic hummingbird-pollinated plant families have different evolutionary histories. For example, in Gesneriaceae, an important hummingbird food source, phylogenetic composition in Central America and the Andes are similar (i.e., dominated by *Columnea*, *Drymonia*), while the Atlantic Forest has distinct clades (*Nematanthus*,

Sinningia) (Clark et al. 2012, Serrano-Serrano et al. 2017). In hummingbirds, the Brilliant and Coquette clades originated in the Andes and are less speciose in both Central America and the Atlantic Forest (Bleiweiss 1998, McGuire et al. 2014). The Mountain Gem and Bee clades are hypothesized to have originated in Central and North America.

Finally, land-use change is a pervasive threat to tropical montane diversity and can influence the network structure and community persistence (Guevara et al. 2023a). Specialist hummingbird species with distinct morphological traits are often lost when landscapes are transformed from native forest habitat to more open habitats dominated by cattle grazing and agriculture (Hadley et al. 2018, Tinoco et al. 2018). Nonetheless, overall hummingbird richness often remains similar across land-use gradients because specialist species are often replaced by generalists (Maruyama et al. 2019, Morrison and Mendenhall 2020, Carranza-Quiceno et al. 2024). From a plant perspective, transformed landscapes often still host some native vegetation and ample sunshine in these more open areas can increase flower abundance. Further, ornamental plants are often planted in hedgerows and open areas, and many of these have flowers that are visited by hummingbirds. Thus, by changing the composition of both hummingbirds and plants, land use change can influence network structure and community stability. We present data collected in pristine and degraded sites in one of our study regions, Ecuador.

Class I. Data Set Descriptors

A. Data set identity:

Plant–hummingbird interactions, floral abundance, and floral traits for three altitudinal gradients in the Americas

B. Data set identification code:

- Cameras_data_all.txt
- Interactions_data_all.txt
- Site_metadata_all.txt
- Transect_data_all.txt
- Plant_traits.txt
- Herbarium_data.txt
- Summary_statistics.txt
- Metadata.xls
- Merging_atlantic_pollination.R
- DarwinCorePPI_compliant.zip

C. Data set description:

1. Originators:

Catherine H. Graham. Spatial Evolutionary Ecology Group. Swiss Federal Research Institute WSL. Zürcherstrasse 111, 8903 Birmensdorf, Switzerland.

Tatiana Santander G. Aves y Conservación/BirdLife in Ecuador, Quito, Ecuador.

María A. Maglianesi. Escuela de Ciencias Exactas y Naturales, Universidad Estatal a Distancia, San José, Costa Rica.

Isabela Galarda Varassin. Laboratório de Interações e Biologia Reprodutiva, Universidade Federal do Paraná, Curitiba, Brazil.

2. Abstract:

Plant–animal interactions result in many ecosystem services, such as pollination. In the western hemisphere, and especially the Neotropics, hummingbirds are important pollinators for thousands of plant species, yet there is much to be learned about the spatial and temporal variation in mechanisms influencing these interactions. We collected an extensive dataset from 39 sites across elevation gradients spanning 4.7 to 3,522 meters above sea level in three regions, the Pacific slope of the Talamanca Mountain range in central-southern Costa Rica, the Chocó-Andean region of northwestern Pichincha province in Ecuador, and the Atlantic Forest in southeastern Brazil. Because these regions have very different biogeographic histories, comparing them allows us to identify general patterns of interactions. Data on flower abundance and plant–hummingbird interactions were gathered monthly for 1.5 to 2 years along 1.5 km transects situated along an elevation gradient in each country during 2017 and 2024. Trait data for flowers was also measured. This effort resulted in 95,824 pairwise interactions between 715 plants and 79 hummingbird species, and also 873 interactions with another 12 bird species in Ecuador. These data are being used to develop a predictive framework on species interactions necessary to understand and conserve biodiversity and ecosystem services. These data also provide detailed information on plants important for hummingbird conservation which have, for example, been used to guide restoration projects. The data are available for reuse under CC BY 4.0 licensing. Please cite the data release on Zenodo and this data paper when reusing the dataset.

D. Key words/phrases: Atlantic Forest-Brazil, Costa Rica, Neotropics/Tropical Andes, Chocó region-Ecuador, Floral traits, Hummingbirds, Plant–animal interaction, Pollination, Nectar-feeding, Networks, Elevation gradient, Habitat disturbance

Class II. Research origin descriptors

A. Overall project description

1. Identity: Compilation of data taken during two years along elevational gradients in Ecuador, Costa Rica and Brazil, including information about flowering plant abundance, flower traits and plant–hummingbird interactions.
2. Originators: Same as above
3. Period of study: Ecuador: Monthly sampling from January 2017 to June 2024 in undisturbed sites, and every two months from December 2017 to December 2019 in disturbed sites; Costa Rica: Monthly sampling from June 2019 to June 2021; Brazil: Monthly sampling from December 2019 to May 2022 (with gaps due to Covid)

4. Objectives:
    - Gather data on plant–hummingbird interactions across space and time in each of three biogeographic regions with distinct evolutionary histories (Ecuadorian Andes, Costa Rica, Brazilian Atlantic Forest);
    - Quantify plant and hummingbird species, plant trait composition, and network structure along the gradients;

- Quantify changes in interaction beta-diversity (i.e., reflecting both change in species composition, and in interacting partners) across land-use and elevation gradients;
- Evaluate the importance of multiple factors, such as trait-matching, environmental conditions, relatedness and abundance, on species interactions, network structure and interaction beta-diversity;
- Evaluate similarities and differences in species interactions, network structure and beta-diversity across regions to identify generalities

5. Abstract: Same as above
6. Sources of funding: European Research Council (ERC) under the European Union's Horizon 2020 research and innovation program (Grant No 787638), the Swiss National Science Foundation (Grant No 173342), and National Geographic Society (Grant No 9952-16), granted to CHG. National Council for Scientific and Technological Development (CNPq, Grant No 312580/2020-7) and Coordenação de Aperfeiçoamento de Pessoal de Nível Superior - Brasil (CAPES, Finance Code 001, Capes-PrInt Grant No 88887.756715/2022-00) to IGV. Support from Aves y Conservación/BirdLife in Ecuador for use of the vehicle, office space, internet, and accountant's time.

B. Specific subproject description

1. Site description

We collected data across the three biogeographic regions 32 transects in forest areas: 11 transects in Ecuador, 12 transects in Costa Rica and 9 transects in Brazil (Fig. 1). In addition to forest sites, in Ecuador data were collected at 7 sites in human dominated agricultural systems. We selected the sites to cover the elevation gradient in each biogeographical. The

choice also included logistical aspects, such as safe protected/guarded areas for people and cameras, availability of a place to stay, permission to use the property for two years and enough trails to establish 1.5 km transects.

In Ecuador we collected the data in the Chocó region which occurs on the western side in the Pichincha Province (latitude 0°12′ N to 0°10′ S, longitude 78°59′ W to 78°27′ W). The region spanned an elevation range from 776 to 3522 meters in north-western Ecuador. The studied area is characterized by average temperatures ranging between 7-20 °C across sites along the elevation gradient, high relative humidity due to the constant presence of mist, particularly during the evenings (González et al. 2015). Precipitation varied across the year with a rainy season from Oct-May, and dryer season from Jun-Sep (Rodríguez et al. 2019). Four different ecosystems are present in this area corresponding to the Western Cordillera of the Andes: evergreen foothill forest (300-1400 m a.s.l.), low evergreen montane forest (1400-2000 m a.s.l.), evergreen montane forests (2000 to 3100 m a.s.l.), and high evergreen montane forest (3100-3600 m a.s.l.) (Ministerio del Ambiente del Ecuador 2013).

Eleven sites were located in preserved mature and secondary forest spread across the elevation gradient but roughly fell into four elevation bands (800-1500, 1501-2200, 2200-2900, 2900-3500 m a.s.l.). Additionally, seven sites in human dominated systems were sampled. These sites consist of formerly forest areas converted into cattle farms characterized by remnants of forest embedded within a pasture matrix (i.e., cattle farms dominated by pastures with hedgerows, live fences, and small forest patches embedded).

In Costa Rica, we collected field data in central-southern Costa Rica on the Pacific slope of the Talamanca Mountain Range. The region of data collection ranged in elevation from 636

m a.s.l. to 3.224 m a.s.l. The average annual temperature in this area is 22-24 ° C in the lower sites and 10-12 ° C in the higher elevation sites, while the range of annual precipitation is 2500-3000 and 3000-4000 mm, respectively. The life zones within this gradient are tropical premontane wet forest, tropical premontane rain forest, tropical lower montane wet forest, tropical lower montane rain forest and tropical montane rain forest (Holdridge 1967).

The 12 study sites were located roughly in four elevation bands (three sites per band) along this gradient. Elevation bands were low (600 to 800 m a.s.l.), mid (1100 to 1500 m a.s.l.), mid-high (1700 to 2200 m a.s.l.) and high (2700 to 3200 m a.s.l.).

In Brazil, we collected data in the Southeastern region, from the sea level along the slopes of the Atlantic forest of the Serra da Bocaina and Serra da Mantiqueira. Nine sites were located in three different elevation bands: low (4 to 170 m a.s.l.), mid (977 to 1201 m a.s.l.), and high elevation (2000 to 2271 m a.s.l.). From the lower to higher bands, the mean annual temperature is 21.9 °C, 16.8 °C, and 11.4 °C, respectively, whereas the mean annual precipitation is 2517 mm, 2120 mm, and 2200 mm, respectively (Instituto Nacional de Meteorologia 2015). The life zones are subtropical lower montane moist forests in lower and mid bands and tropical lower montane moist forests in the higher elevation (Holdridge 1967, de Lima et al. 2022).

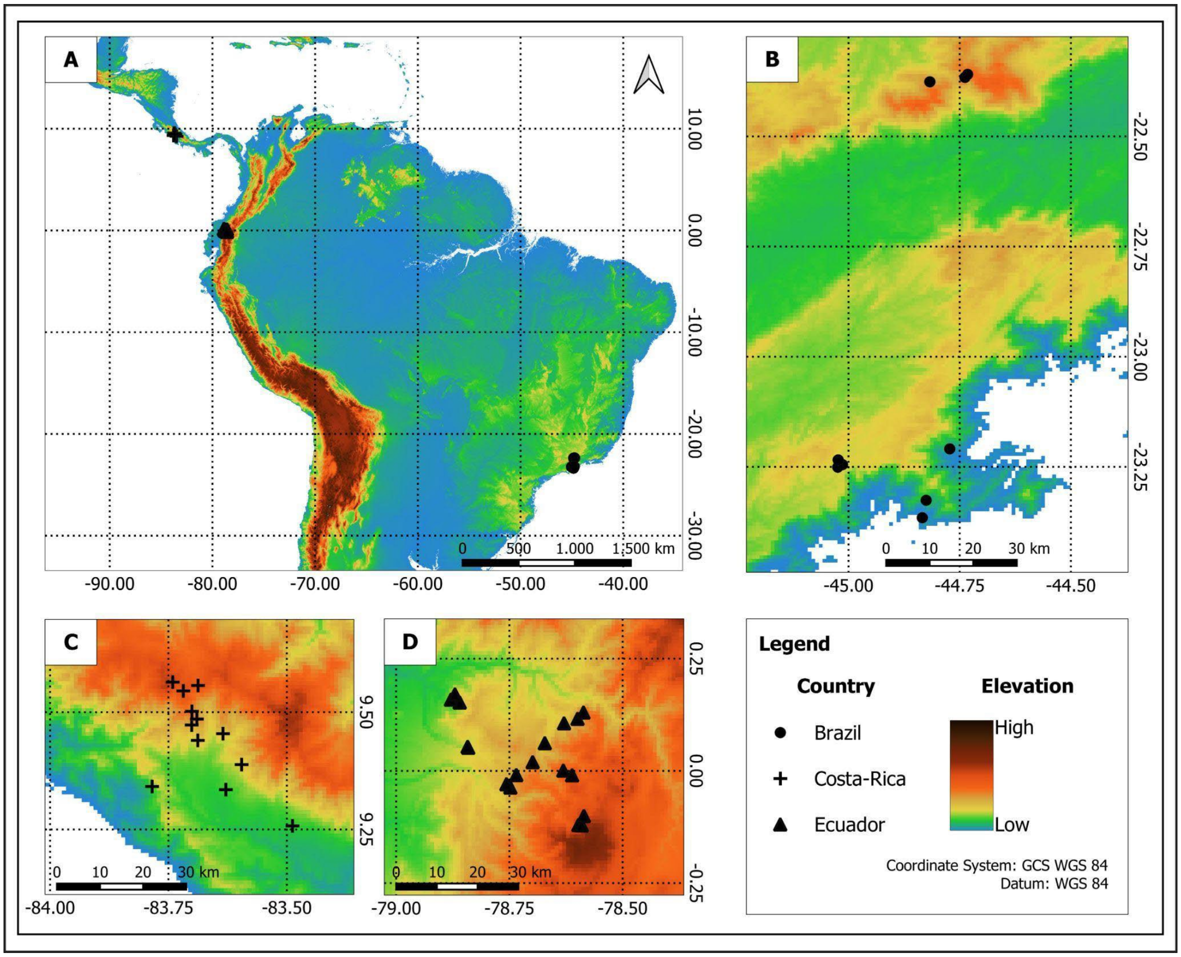

Figure 1. Distribution of field sampling locations (A) along the elevation gradients in the three mountain systems of Brazil (B), Costa Rica (C), and Ecuador (D).

2. Sampling design

We established 1.5 km by 10 m transects in each site using existing trails. At the beginning of each transect sampling we recorded: date and time. Interaction and floral abundance data were collected monthly in undisturbed sites and every two months in disturbed sites in Ecuador. Flower abundance sampling (below) was only done on days with good visibility and limited rain. In some cases, cameras were moved on days with some rain.

3. Research methods

a. Field/laboratory

Flower abundance

We walked the transects at a slow pace (approx. 500 m per hour; 3 hours total) in order to observe flowers. In some cases, where there was high flower abundance the pace was slower and the sampling took 4 to 5 hours. The field technician scanned an area of 5 meters on either side of the transect for hummingbird flower resources. A flower was considered a hummingbird resource when it had a hummingbird syndrome (Faegri and van der Pijl 1979): brightly colored flowers - purple, red, orange - with medium to long somewhat narrow corollas), or had been previously observed as visited by hummingbirds by a person or camera. Because hummingbirds visit flowers that do not have a typical hummingbird syndrome (Maruyama et al. 2013, Waser et al. 2018, pers obs.), an effort was made to sample other flowering plants potentially visited by hummingbirds. Other plants visited by hummingbirds were identified either based on direct records in the field by the technician on a transect or by the cameras that were placed on questionable flowers to confirm the plant as a food resource for hummingbirds. All flowering plants with flowers for hummingbirds were identified and a waypoint was taken for each plant using a GPS.

After being identified as a hummingbird resource, the flowers were counted using binoculars. When the plant had fewer than ~100 flowers, all open flowers were directly counted. When the direct counting of flower abundance proved impractical due to a high volume of flowers (>100), we used an estimation method. This involved counting the number of units on the plant, where the unit could be a flower clump or a branch and then counting the number of flowers on 5 units. Then, we calculated the final flower abundance by multiplying the average count of flowers per unit by the total number of units on the plant.

To integrate flower census and camera data, we defined a unique campaignID for each continuous sampling period at a site. A new ID was created when camera deployments were separated by more than eight days, and flower censuses within five days before or after that interval were linked to the same campaign. This approach ensured that interaction data could be consistently associated with their local floral context. In undisturbed sites campaigns were monthly, whereas in disturbed sites in Ecuador they were conducted every two months.

Plant hummingbird interactions

We collected interaction data between plants and hummingbirds using time-lapse cameras placed on individual flowers (or flower clumps) along the same transects where flower abundance was sampled. The cameras were placed up to 3 m above the ground, approximately 1 m from the flower (Fig. 2A,B). The flower was framed in order to film an area larger than the hummingbird body (Fig. 2C), and in Costa Rica and Brazil we annotated the number of open flowers in the frame. We used Plot Watcher Pro cameras, which are no longer available on the market (720p Video, smallest time interval 1 frame per second). We set the camera to take an image every second from dawn to dusk. We aimed to record 12 flowering plants every month, chosen to maximize the number of plant species each month as well as represent proportion to their presence on the transect. This was done by placing 6 cameras on the same day as flower abundance was counted on a given transect. These cameras were left in place for 3 days and moved on the afternoon of the 3rd day or morning of the 4th day to 6 new flowers. We could not always meet this target for a variety of reasons including: cameras were moved (by falling branches, curious wild animals or people), technical issues with filming (batteries or memory card malfunctioned or camera was not

correctly set up), and lack of flowers. Further, there were rare instances when we could not find flowers on the transect to place cameras (i.e., not sufficient flowers, flowers too high); in this case we placed cameras outside of the transect but within 25 meters. Note that this means that a filmed plant species is not listed in the abundance data for a given transect/month because the camera was put outside the transect where the floral abundance was sampled. Whenever we detected a problem with the camera (e.g., turned off before expected, camera was moved, out of focus), we noted it in the dataset. Finally, and importantly for network studies, we did not sample all flowers blooming in a given month on a given transect so monthly networks are incomplete.

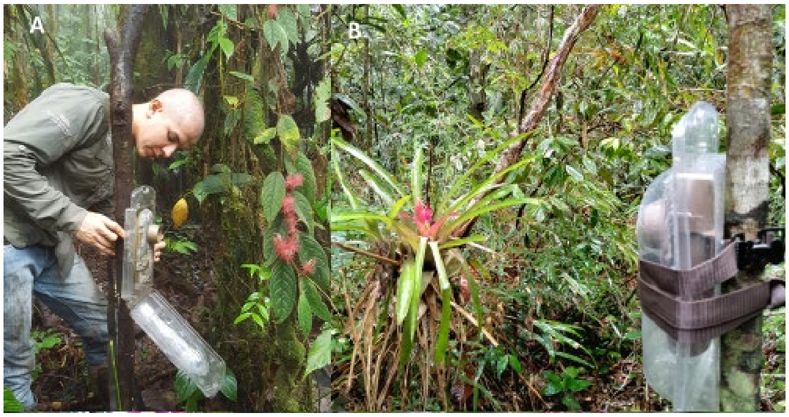

Figure 2. Time-lapse cameras to record plant–hummingbird interactions. (A) installation of camera; (B) positioning of the cameras in front of a *Nidularium* inflorescence (photo credits: A. Nieto).

After three days, the SD card was removed from the camera for processing. All footage was downloaded onto a computer resulting in ~ 43,200 images per camera per day. These images were processed using DeepMeerkat (Weinstein 2015) to identify images with hummingbirds. We inspected the images where the program identified a hummingbird and manually

recorded hummingbird species, sex (depending on species), feeding activity (perching, Fig. 3A-D, vs hoverflying, Fig. 3E-J), and behavior (legitimate visit, Fig. 3, vs. nectar robbing, Fig. 4). This approach minimized reliance on time-consuming flower observations and increased data collection in time and space (Weinstein 2015, Weinstein and Graham 2017). Hummingbird taxonomy followed the GBIF backbone taxonomy (GBIF Secretariat 2025).

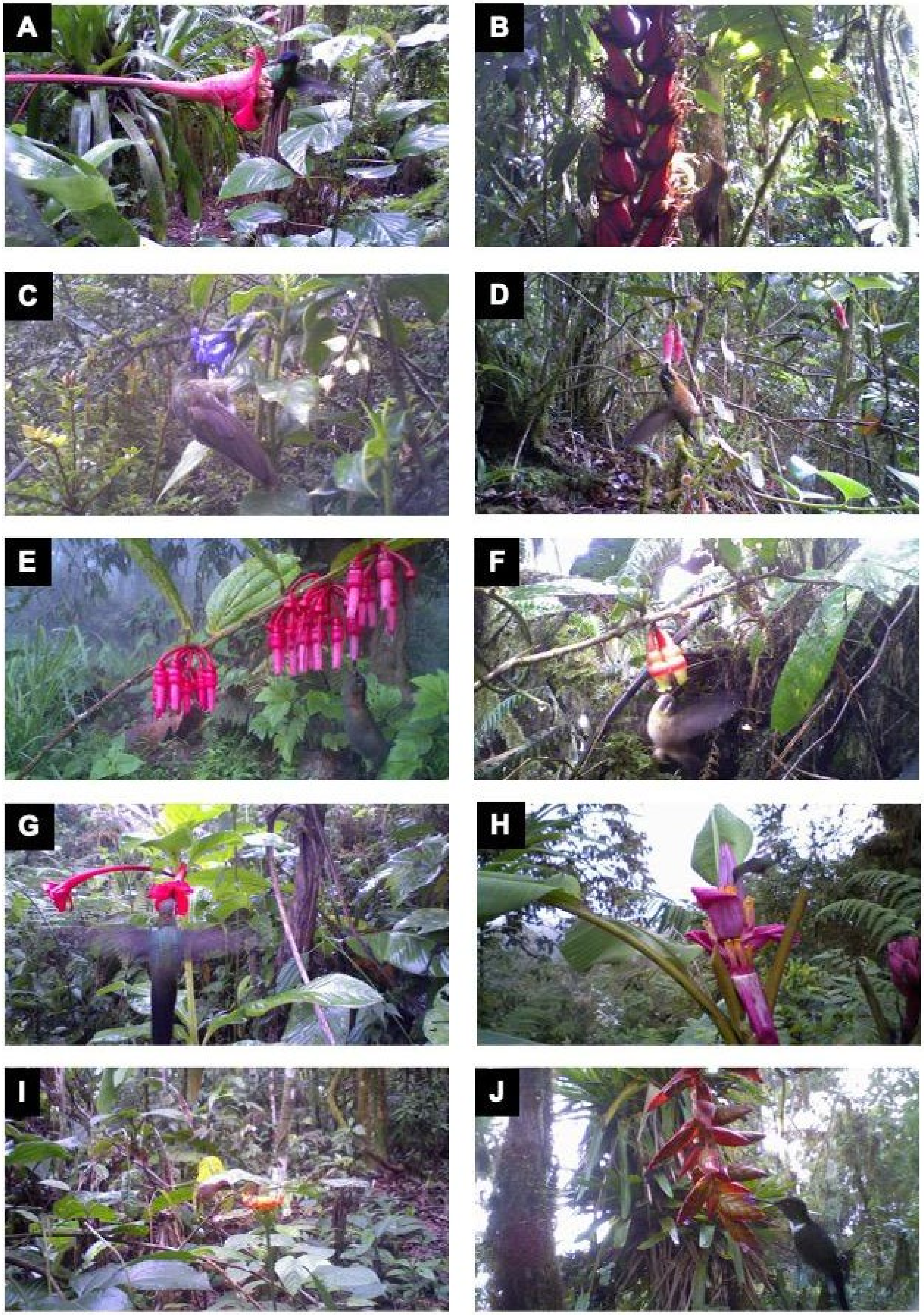
A
B
C
D
E
F
G
H
I
J

Figure 3. Legitimate plant–hummingbird interactions recorded with the time-lapse cameras between (A) *Edmundoa ambigua* (Bromeliaceae) and *Thalurania glaucopis* (photo credit: R. de Oliveira); (B) *Heliconia harlingii* (Heliconiaceae) and *Eutoxeres aquila* (photo credit: A. Nieto); (C) *Palicourea fuchsioides* (Rubiaceae) and *Metallura tyrianthina* (photo credit: T. Santander); (D) *Macleania rupestris* (Ericaceae) and *Lampornis castaneoventris* (photo credit: E. Brenes); (E) *Psammisia ulbrichiana* (Ericaceae) and *Doryfera ludovicae* (photo credit: A. Marcayata-Fajardo); (F) *Psammisia oreogenes* (Ericaceae) and *Adelomyia melanogenys* (photo credit: F. Richter); (G) *Fuchsia macrostigma* (Onagraceae) and *Phaethornis yaruqui* (photo credit: F. Richter); (H) *Musa velutina* (Musaceae) and *Thalurania colombica* (photo credit: E. Guevara); (I) *Gurania makoyana* (Cucurbitaceae) and *Phaethornis striigularis* (photo credit: K. Zuniga); and (J) *Tillandsia complanata* (Bromeliaceae) and *Coeligena torquata* (photo credit: A. Marcayata-Fajardo).

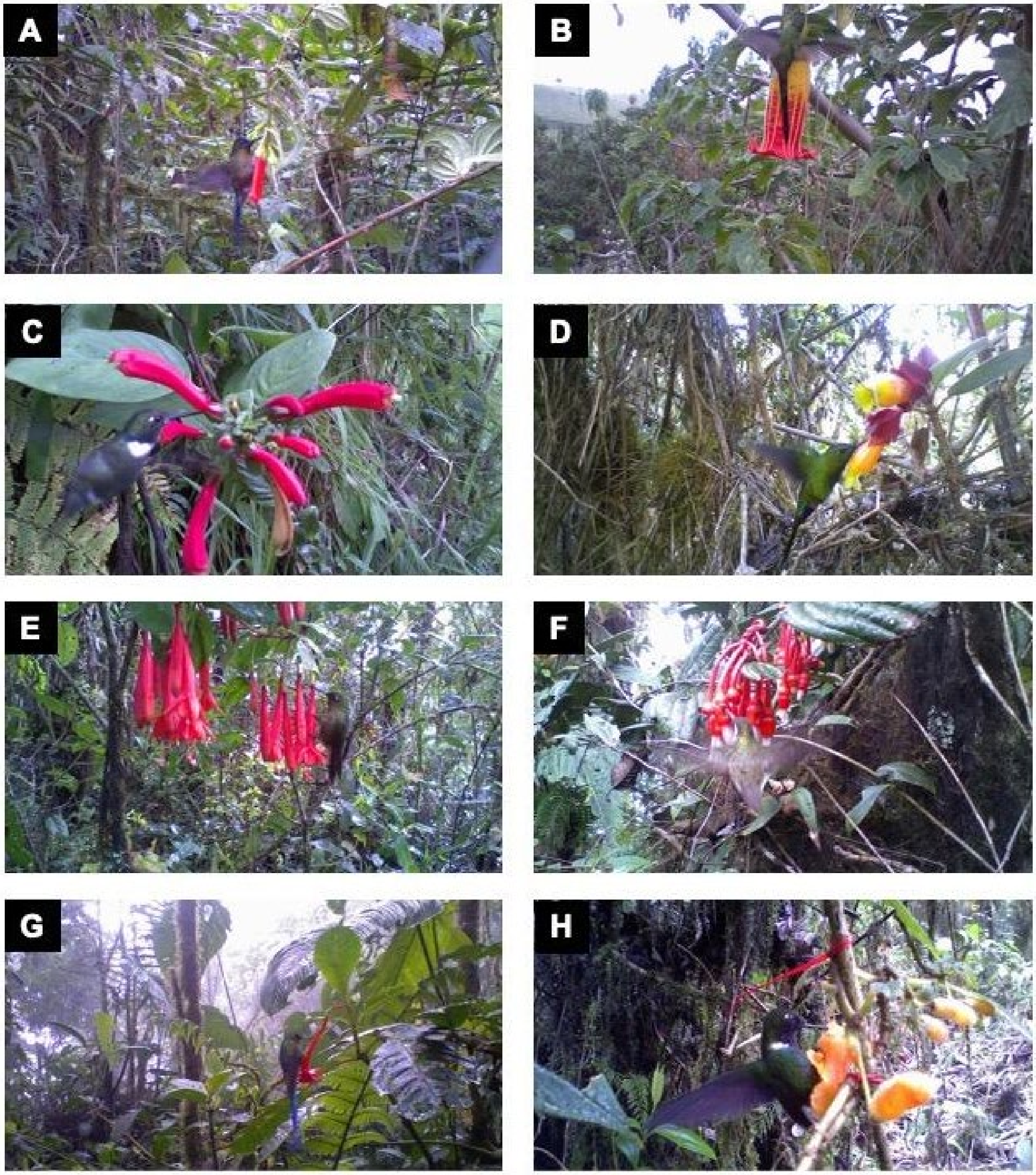

Figure 4. Illegitimate (nectar robbing) plant–hummingbird interactions recorded with the time-lapse cameras between (A) *Macleania bullata* (Ericaceae) and *Aglaiocercus coelestis* (photo credit: F. Richter); (B) *Brugmansia sanguinea* (Solanaceae) and *Lesbia victoriae* (photo credit: E. Guevara); (C) *Centropogon solanifolius* (Campanulaceae) and *Schistes albogularis* (photo credit: H. Beck); (D) *Columnea dielsii* (Gesneriaceae) and *Eriocnemis*

luciani (photo credit: T. Santander); (E) *Fuchsia corollata* (Onagraceae) and *Metallura tyrianthina* (photo credit: A. Marcayata-Fajardo); (F) *Psammisia ulbrichiana* (Ericaceae) and *Adelomyia melanogenys* (photo credit: A. Nieto); (G) *Fuchsia macrostigma* (Onagraceae) and *Aglaiocercus coelestis* (photo credit: F. Richter); and (H) *Columnea strigosa* (Gesneriaceae) and *Heliangelus strophianus* (photo credit: A. Marcayata-Fajardo).

Flower traits

We recorded flower traits for at least five flowers across different individuals, when possible, for each species considered a hummingbird resource plant (see above). This was possible for 76.0% of the species with trait data; however, 6.2%, 2.8%, 5.4% and 9.6% were measured on 4, 3, 2 and 1 individuals; respectively. In total we have flower traits for 665 out of the 926 plant species in the dataset (camera + transect). Thus, 156 filmed plant species are missing traits (25 of these had interactions) and 274 plant species from the transect are missing traits.

We took field photographs to obtain an image of each flower. We first took a picture standing directly in front of the plant, holding the camera at the same level as the flower and framing the main stem section or branch that held the flowers (Fig. 5A). We then removed the flower from the plant to take a scaled close-up photograph of an individual flower. We took photographs with a dorsal (Fig. 5B) and lateral view of the flower. Then, we sectioned the flower longitudinally and took another photograph of the flower in a lateral view (Fig. 5C). From the photos, we measured traits known to influence plant–hummingbird interactions, considering three aspects: visibility, access to resources, and reproduction.

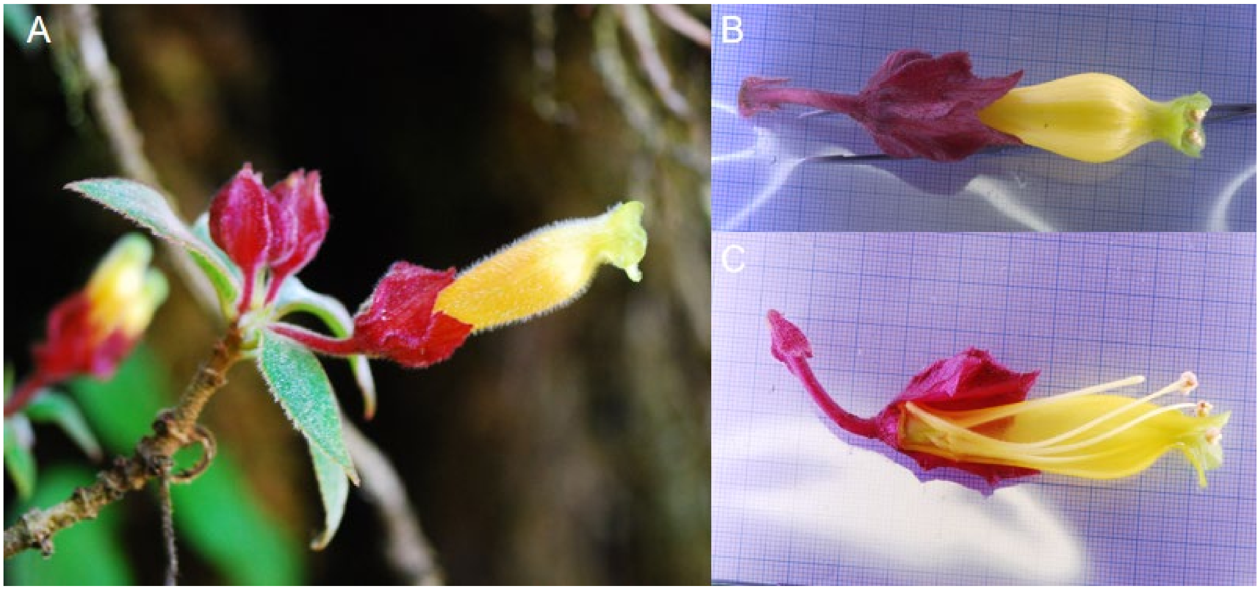

Figure 5. Field photographs of *Columnea dielsii* (Gesneriaceae). (A) Branch of a flowering plant; (B) intact flower; (C) lateral section (photo credits: F. Tobar).

Visibility traits are those associated with hummingbird attraction. We recorded which plant organ had bright colors: calyx and/or corolla (Fig. 6A, C) or accessory structures such as bracts (Fig. 6B), leaves (Fig.6C) and/or stems (Fig. 6A).

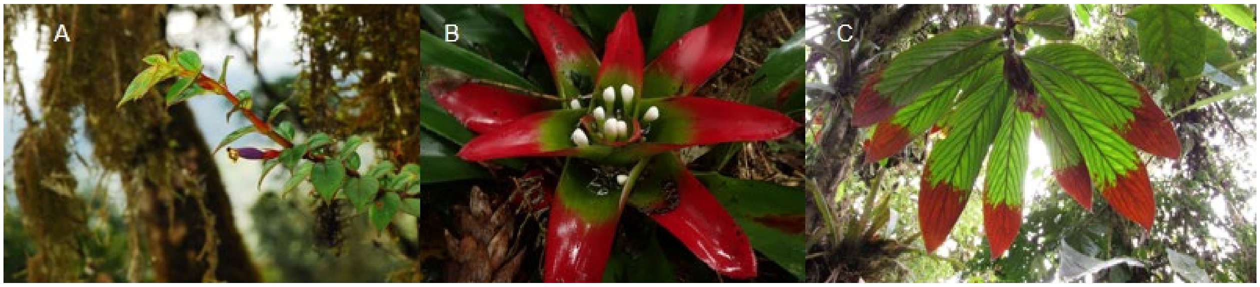

Figure 6. Visibility traits: coloured organs. (A) *Columnea katzensteiniae* (Gesneriaceae) with colored flowers and stems (photo credit: F. Tobar); (B) *Nidularium innocentii* (Bromeliaceae) with colored bracts (photo credit: R. de Oliveira); (C) *Columnea medicinalis* (Gesneriaceae) with colored leaves (photo credit: F. Tobar).

Access traits are those associated with the access of pollinators to food resources. We recorded flower position: axillar (Fig. 7A, C, E), terminal (Fig. 7B, F), caulinar (Fig. 7D); Flower orientation: pendular (Fig. 7A, F), erect (Fig. 7B, C), horizontal (Fig. 7E); flower symmetry: actinomorphic (Fig. 8A), zygomorphic (Fig. 8B), asymmetric (Fig. 8C); occurrence of resupination: non-resupinated (Fig. 8D) and resupinated (Fig. 8E) flowers; corolla fusion: gamopetalous and dialipetalous; corolla curvature (Fig. 9A), tube length (Fig. 9B), corolla width lateral (Fig. 9B) and, for zygomorphic flowers, corolla width dorsal (Fig. 9C).

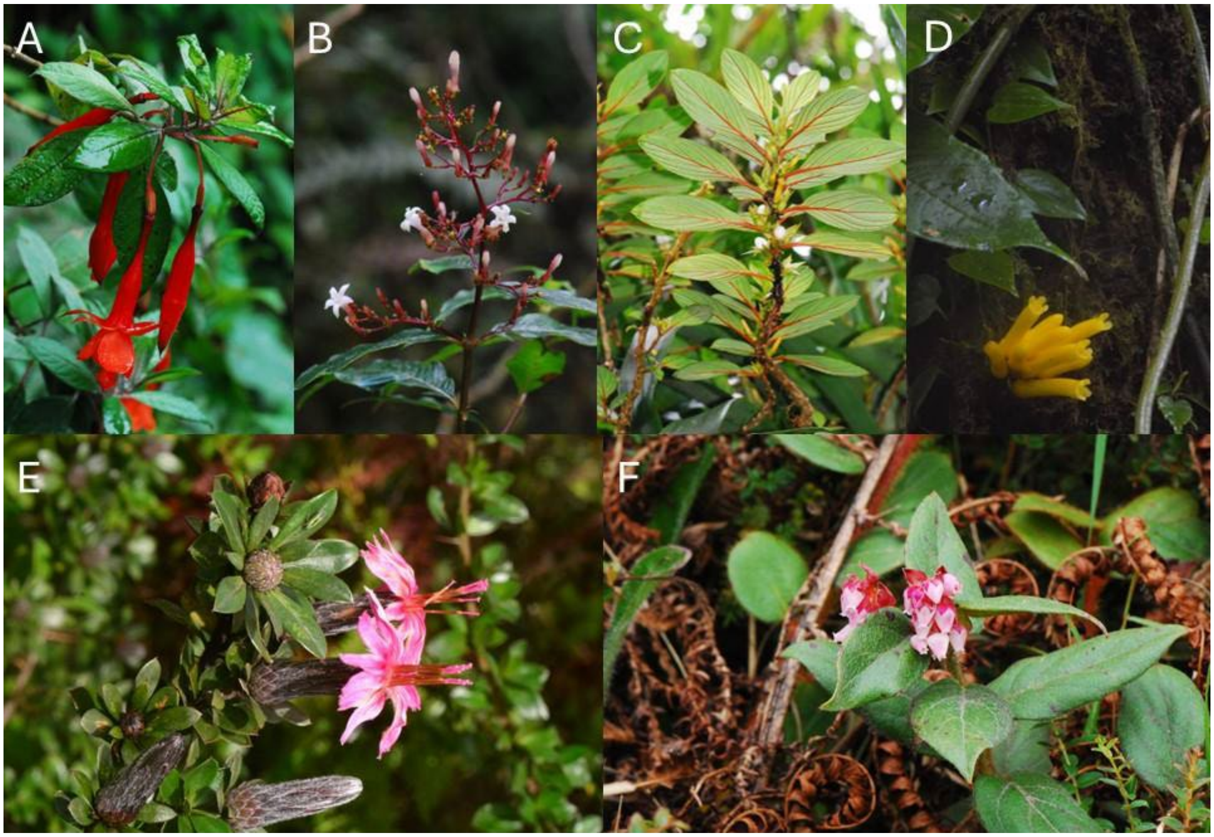

Figure 7. Access traits: flower position and orientation of different taxonomic groups. (A) *Fuchsia ayavacensis* (Onagraceae), with axillary pendulous flowers emerging in the leaf axils of the lower part of the branch; (B) *Palicourea calothyrsus* (Rubiaceae), with terminal horizontal flowers; (C) *Columnea angulata* (Gesneriaceae), with axillary erect flowers emerging below the leaves; (D) *Schlegelia sulphurea* (Schlegeliaceae), with caulinar flowers; (E) *Barnadesia spinosa* (Asteraceae), with axillary horizontal flowers; and (F) *Gaultheria glomerata* (Ericaceae), with terminal pendulous flowers (photo credits: F. Tobar).

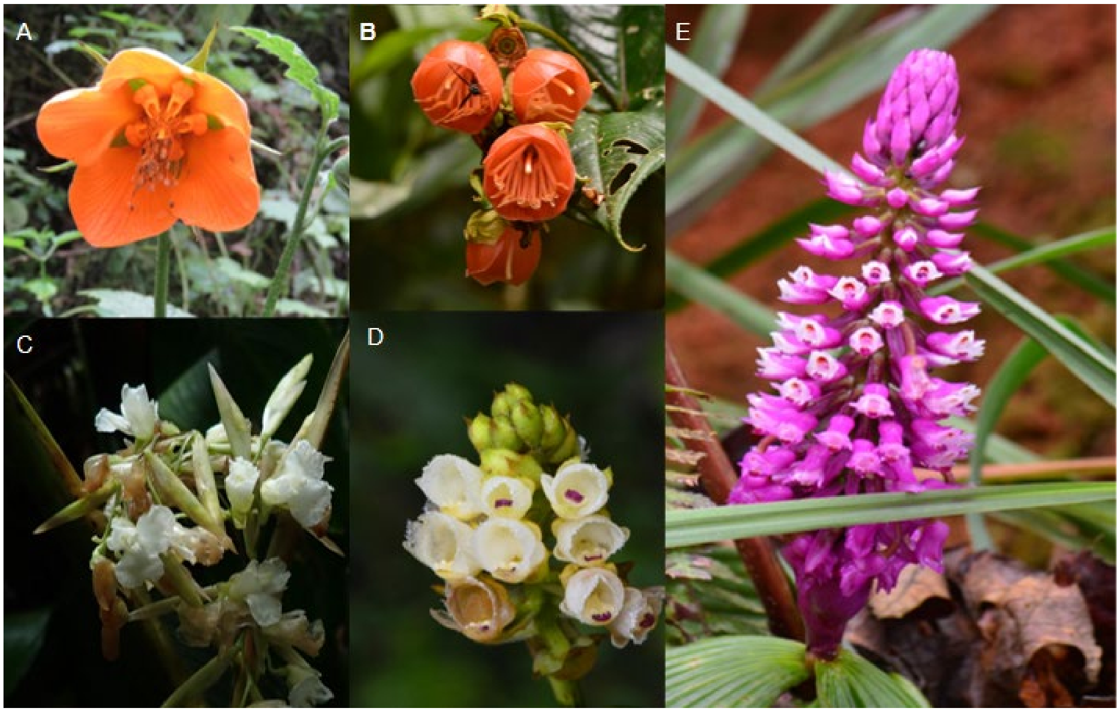

Figure 8. Access traits: flower symmetry and resupination. (A) *Nasa grandiflora* (Loasaceae) with actinomorphic flowers (photo credit: T. Santander); (B) *Meriania tomentosa* (Melastomataceae) with zygomorphic flowers (photo credit: F. Tobar); (C) *Stromanthe papillosa* (Marantaceae) with asymmetric flowers (photo credit: R. de Oliveira); (D) *Elleanthus aurantiacus* (Orchidaceae) with non resupinated flowers (photo credit: F. Tobar); and (E) *Elleanthus amethystinoides* (Orchidaceae) with resupinated flowers (photo credit: F. Tobar).

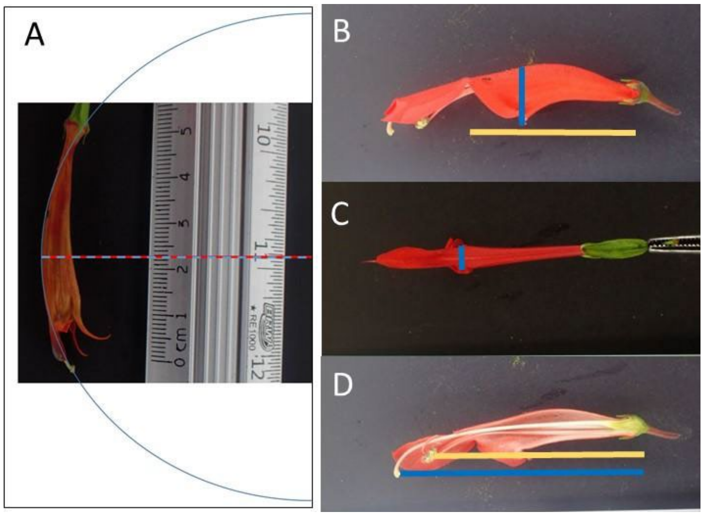

Figure 9. Access and reproductive traits: (A) corolla curvature; (B) tube length (blue), corolla width lateral (yellow) and; (C) for zygomorphic flowers, corolla width dorsal; (D) style (blue) and stamen (yellow) lengths (photo credits L. Romanowski).

Reproductive traits are those associated with reproduction or pollen deposition such as sexual system, style and stamen length (Fig. 9D), the position of the anthers: ventral (Fig. 10A), dorsal (Fig. 10B), radial (Fig. 10C), and anther dehiscence: pores (Fig. 10C, D), valvalte (Fig. 10E), longitudinal slits, transverse slits (Fig. 10F), and rimose, for species with pollinia (Fig. 10G). Among reproductive traits, we described the gender distribution in the flower or plant as cosexual, dioecious, or monoecious; the occurrence of distyly, that is the presence of floral polymorphism comprising two different floral phenotypes, was scored as yes or no; and the timing of maturation of male and female reproductive organs in cosexual flowers, as homogamy (when both male and female reproductive organs are mature at the same time) or as protandry (when male organs mature first).

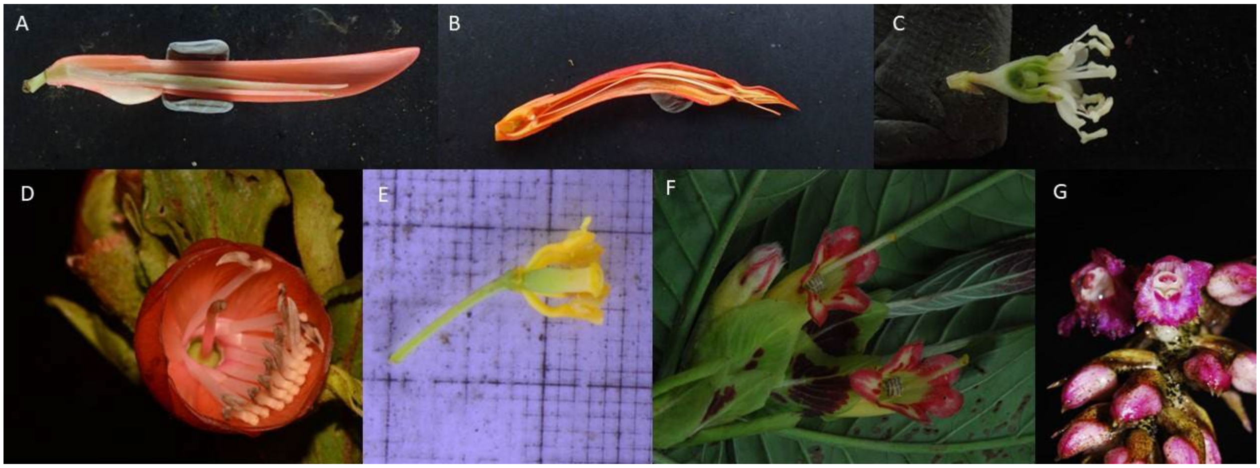

Figure 10. Reproductive traits: anther position and dehiscence. Position: (A) *Erythrina costaricensis* (Fabaceae), with ventral anthers (photo credit: N. Chaves-Elizondo); (B) *Aphelandra lingua-bovis* (Acanthaceae), with dorsal anthers (photo credit: N. Chaves-Elizondo); (C) *Miconia schnellii* (Melastomataceae), with radial anthers (photo credit: N. Chaves-Elizondo). Dehiscence: (D) *Meriania tomentosa* (Melastomataceae) with poricidal dehiscence (photo credit: F. Tobar); (E) *Berberis paniculata* (Berberidaceaae), with valvate anthers (photo credit: F. Tobar); (F) *Columnea medicinalis* (Gesneriaceae) with longitudinal slits (photo credit: F. Tobar); and (G) *Elleanthus amethystinoides* (Orchidaceae), with rimose anthers (photo credit: F. Tobar).

We recorded both flower shapes and types. Flower shape is a functional description either from single flowers or inflorescences related to pollination. We classified it in seven shapes, adapting from Faegri & Pijl (1979) in order to to match the classification used in the Atlantic Pollination dataset (Iamara-Nogueira *et al*., 2021): bell (Fig. 11A), brush (Fig. 11B), dish (Fig. 11C), flag (Fig. 11D), gullet (Fig. 11E), spurred (Fig. 11F), and tube (Fig. 11 G, H, I). Flower type, on the other hand, reports the morphology of a flower, and was classified in eight main types: bilabiate (Fig. 11H), campanulate (Fig. 11E, F), funnelform (Fig. 11A),

hypocrateriform (Fig. 11G), ligulate (Fig. 11C) and tubular (Fig. 11B, D). Flower shape and type often converge but may also differ, as in *Barnadesia spinosa* (Fig. 11C) and *Calliandra houstoniana* (Fig. 11 B). In *Barnadesia spinosa* the flower type is ligulate, but the flower shape is dish, since the attraction unit is the inflorescence and it has radial symmetry and their stamens and anthers are fully exposed in the center of the flower or inflorescence (Faegri & van der Pijl 1979). In *Calliandra houstoniana* the flower type is tubular, but the flower shape is brush, since the attraction unit is the inflorescence and it has the anthers and stigmas completely exposed. The corolla has radial symmetry and is bent downward or very small (Faegri & van der Pijl 1979).

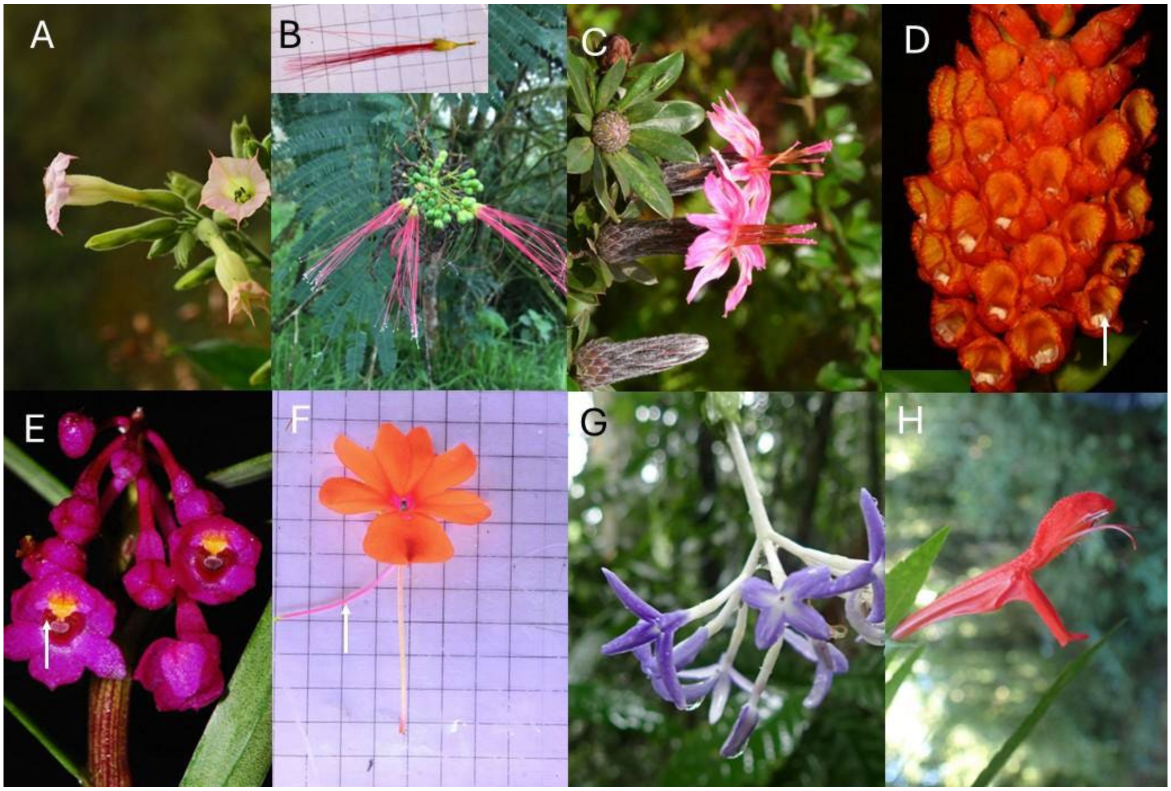

Figure 11. Flower shapes and types: (A) *Nicotiana tabacum* (Solanaceae), a bell-shaped flower and a funnelform flower type (photo credit: F. Tobar); (B) *Calliandra houstoniana*

(Fabaceae), a brush-shaped inflorescence and a tubular flower type (photo credit: F. Tobar); (C) *Barnadesia spinosa* (Asteraceae), with a dish-shaped inflorescence and a single ligulate flower (photo credit: F. Tobar); (D) *Elleanthus discolor* (Orchidaceae) flag-shaped flower with the reproductive organs in the lower portion of the flower (arrow) and a tubular flower type (photo credit: F. Tobar); (E) *Epidendrum mesogastropodium* (Orchidaceae) gullet-shaped flower with the reproductive organs in the upper portion of the flower (arrow) and a campanulate flower type (photo credit: F. Tobar); (F) *Impatiens walleriana* (Balsaminaceae) spurred-shaped flower with a calcar (arrow) and a campanulate flower type (photo credit: F. Tobar); (G) *Faramea paratiensis* (Rubiaceae), tube-shaped flower with landing area and narrow tube and a hypocrateriform flower type (photo credit A. Caron); (H) *Salvia balaustina* (Lamiaceae), a tube-shaped flower and a bilabiate flower type (photo credit R. de Oliveira).

c: <u>Taxonomy and systematics (plant species identity and vouchers)</u>

We collected the plant species recorded for flower abundance and interactions (below). The vouchers from Ecuador were deposited at the Herbaria Catholic University of Ecuador (QCA) and Catholic University-Ibarra campus (PUCE-SI); from Brazil at the herbarium of Botany Department (UPCB - Universidade Federal do Paraná), Herbário Dom Bento José Pickel (SPSF- Instituto Florestal), and Herbário Dimitri Sucre (RB - Jardim Botânico do Rio de Janeiro); and from Costa Rica at the Luis A. Fournier Herbarium (USJ) at the Universidad de Costa Rica

The species' identity was checked by a local expert and, when not it was not possible to identify a given species, by an expert on the taxonomic group. Plants in Ecuador were identified by Francisco Tobar (all groups), John Clark (Gesneriaceae), Alvaro Peréz

(Acanthaceae and Rubiaceae), Eric Hagsater (Orchidaceae), Dario Garcia (Ericaceae), Diana Fernandez (Melastomataceae); in Brazil, Cintia Kameyama (Acanthaceae), Daniela Zappi (Rubiaceae), Luiz Henrique Fonseca (Bignoniaceae), Maria Fernanda Calió (Rubiaceae), and Miriam Kaehler (Bignoniaceae and Bromeliaceae), in Costa Rica, Nelson Chaves Elizondo. In some cases new species were described (Clark et al. 2021, Clark and Tobar 2021, Suarez et al. 2021, Tobar et al. 2022g). Plant taxonomy followed the GBIF backbone taxonomy (GBIF Secretariat 2025). With the field photographs we produced plant guides to be used in field expeditions and to provide for each site the list of plants visited by hummingbirds for Ecuador only (Tobar et al. 2020a, 2020b, 2021, 2022a, 2022b, 2022c, 2022e, 2022d, 2022f).

d. Permit history

All field activities were conducted under the appropriate research and collection permits issued by the relevant national authorities:

- Ecuador: Ministry of Environment in Ecuador – permit Nº 0162019ICFLOFAUDNB/MAE.
- Costa Rica: Sistema Nacional de Áreas de Conservación (SINAC) – permits R-SINAC-PNI-ACLAP-017-2020, R-SINAC-PNI-ACLAP-048-2021, SINAC-ACC-PI-R-059-2019, and SINAC-ACC-PI-R-078-2021.
- Brazil: Instituto Chico Mendes de Conservação da Biodiversidade (ICMBIO) – permits Nº 68624-2, 74216-1, and 69412-1; Instituto de Pesquisas Ambientais (IPA) – permit SMA Nº 260108 – 005.133/2019; and Sistema Nacional de Gestão do Patrimônio Genético e do Conhecimento Tradicional Associado (SISGEN) – access registration Nº A81367A.

Class III. Data set status and accessibility

A. Status

1. Latest update: 8th of September 2025
2. Latest archive date: 8th of September 2025
3. Metadata status: 8th of September 2025. Status: Complete.
4. Data verification: Complete

B. Accessibility

1. Storage location: The complete data set and R scripts are available as Supporting Information to this data paper publication in *Ecology*. The data are also available in Zenodo in Santander et al. (2025) at https://doi.org/10.5281/zenodo.17092717.
2. Contact person: Catherine H. Graham. Spatial Evolutionary Ecology Group. Swiss Federal Research Institute WSL. Zürcherstrasse 111, 8903 Birmensdorf, Switzerland. E-mail: catherine.graham@wsl.ch. Phone: +41 44 739 2361.
3. Copyright restrictions: The data are available for reuse under CC BY 4.0 licensing. Please cite the data release on Zenodo and this data paper when reusing the dataset.
4. Proprietary restrictions: None
5. Costs: None

Class IV. Data structural descriptors

A. Data set file

1. Identity:

- Cameras_data_all.txt
- Interactions_data_all.txt
- Site_metadata_all.txt
- Transect_data_all.txt
- Plant_traits.txt

- Herbarium_data.txt
- Summary_statistics.txt
- Metadata.xls
- Merging_atlantic_pollination.R
- DarwinCorePPI_compliant.zip

2. Size: 29.2 MB, 10 items.
3. Format and storage mode: ASCII .txt files, with comma-separated values. R scripts.
4. Header information: None
5. Alphanumeric attributes: Mixed
6. Special characters/fields: None
7. Authentication procedures: None

B. **Variable Information**

The dataset is organized into five spreadsheets: (1) cameras, (2) interactions, (3) sites, (4) transects, and (5) plant traits, and (6) herbarium records (Fig. 12). The camera table (Table 1) documents each camera deployment, including its unique ID (waypoint), site, start and end date and time, sampling duration, filmed plant species, and geographic coordinates. The interactions table (Table 2) records all bird–flower interactions captured by the cameras, including the bird species, date and time of the event, and type of behavior; records can be linked to the camera table through the waypoint column. The sites table (Table 3) provides metadata on each site, such as country, site name, latitude and longitude, elevation, area, forest type, and additional habitat descriptors. The transect table (Table 4) contains the flower census data, including plant species identity and floral abundance, along with the campaignID used to link census records to corresponding camera deployments. The plant

traits table provides the plant identity and a series of continuous and categorical floral traits for the species present in the transect and/or camera tables (Table 5). The herbarium table (Table 6) lists the specimens deposited in herbaria, including species identification, the person responsible for the collection and identification of the specimen, the herbarium where each specimen is stored, and the unique catalog identifier assigned to each sample.

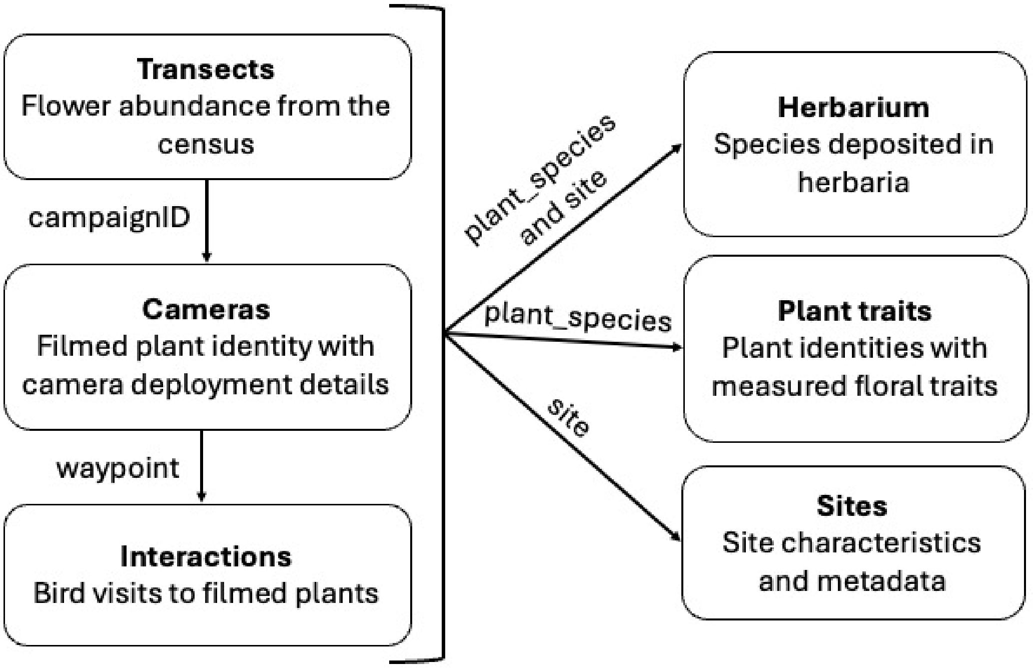

Fig. 12 - Overview of the dataset structure. Each box represents a table containing information on transects, cameras, bird interactions, floral traits, and sites. The labels beside the connecting arrows indicate the column used to merge the tables.

Alongside these original data tables, we provide a Darwin Core + Plant–Pollinator Interactions compliant version of the dataset (Salim et al. 2022) and an R script that performs the transformation. This standardized representation facilitates machine readability, interoperability, and data merging across studies, while the accompanying script ensures

transparency and reproducibility by allowing users to regenerate or adapt the transformation process as needed.

B.1 Table 1. Camera information

Variable identity	Variable definition	Unit/Levels of measurement
waypoint	Unique camera ID	Character
site	Site name, additional information are on file Site.txt	Character
plant_scientificName	Full plant species scientific name according to the GBIF backbone taxonomy, with authorship and date information	Character
plant_usageKey	The GBIF taxonKey at the lowest taxonomic level, which is a unique identifier assigned by GBIF to a taxon in its backbone taxonomy.	Numeric
plant_species	Latin name of the plant species according to the GBIF taxonomy. If plant was not identified at species level, an ID of the unknown species is written, such as "Bromelia sp1"	Character
plant_genus	Plant taxonomy according to the GBIF backbone taxonomy	Character
plant_family		Character
plant_order		Character
start_date	Date when the camera started recording	YYYY-MM-DD
end_date	Date when the camera finished recording	YYYY-MM-DD
start_time	Time of day when the camera started recording	HH:MM:SS
end_time	Time of day when the camera finished recording	HH:MM:SS
duration_sampling_hours	Total hours of sampling, calculated as the time difference between start and end time. This time duration only considers daylight hours, as the cameras were programmed to turn on at dawn and off at dusk	hours
duration_from_pics	Approximate total hours of sampling obtained from the time difference between the first and last interaction pictures,	hours

Variable identity	Variable definition	Unit/Levels of measurement
	considering the hours of daylight. This field can be useful if the information from duration_sampling_hours is missing	
longitude_waypoint	Longitude of the waypoint associated to the camera	WGS 84, EPSG:4326
latitude_waypoint	Latitude of the waypoint associated to the camera	WGS 84, EPSG:4326
elevation_waypoint	Elevation of the waypoint associated to the camera	m above sea level
time_waypoint	The date and time when the waypoint was recorded in the GPS	YYYY-MM-DD HH:MM:SS
camera_height_cm	Height above the ground where the camera was positioned	meters
camera_flowers_count	Number of open flowers in the frame. Not available for data from Ecuador	count
camera_problem	Categorical classes indicating whether there was a problem with the camera	Levels: "no" - no problem with the camera; "yes" - there was a problem with the camera, "maybe"- not clear whether the camera recorded as expected, and "flower_problem" - the flower was covered by a leaf or flowers closed or felt during the sampling. NA if it was not specified.
campaignID	Unique identifier for each sampling campaign at a site, assigned based on camera deployments (a new consecutive ID is created when deployments are >8 days apart).	Character (site name + sequential number)

Variable identity	Variable definition	Unit/Levels of measurement
country	Country where the data were collected	Brazil; Costa-Rica; Ecuador

B.1 Table 2. Interactions (plant–bird)

Variable identity	Variable definition	Unit/Levels of measurement
waypoint	Unique camera ID	Character
site	Site name, additional information are on file Site.txt	Character
date	Date of the picture	YYYY-MM-DD
time	Time when the picture was recorded	HH:MM:SS
bird_scientificName	Full bird species scientific name according to the GBIF backbone taxonomy, with authorship and date information	Character
bird_usageKey	The GBIF taxonKey at the lowest taxonomic level, which is a unique identifier assigned by GBIF to a taxon in its backbone taxonomy.	Numeric
bird_species	Latin name of the bird species detected according to GBIF taxonomy. Can be NA if species was not identified	Character
bird_genus	Bird taxonomy according to the GBIF	Character
bird_family		Character
bird_order		Character
bird_sex	The sex of the bird if specified	“male”, “female”, or NA if it was not specified.
interaction	Whether the bird was recorded interacting with the flower or not	"yes" for pictures where the bird was seen engaging with the flower; "no" for when the bird in the picture was not engaging with the flower; NA if it was not

Variable identity	Variable definition	Unit/Levels of measurement
		specified.
feeding_activity	The way the bird interacted with the flower if specified	“hoverflying” for bird interacting with flowers while flying; “perching” for bird interacting with flowers while perched; "perching,hoverflying" for a combination of both; “no_feeding” if the bill of the bird was not touching the flower, or NA if it was not specified.
nectar_robbing	The foraging behavior of the bird in terms of legitimate feeding, where the reproductive parts of the plant touched the bird, or nectar robbing where the bird pierced a hole in the flower base or used an existing hole to extract nectar	“yes” indicates nectar robbing, where the bird used a hole at the base of the flower to extract nectar; “no” indicates a legitimate interaction; “maybe” indicates possible nectar robbing that could not be conclusively determined from the picture; “thief” indicates nectar robbing without the use of a hole (e.g., feeding from nectaries of *Marcgraviastrum gigantophyllum*); “not_interacting” applies when the bird was visible in the picture but not observed interacting with the flower; and NA is used when no information was specified.

Variable identity	Variable definition	Unit/Levels of measurement
		Note that in data from Ecuador, "no" was also used in cases where the robbing status could not be fully determined because the bird was positioned behind the flower, meaning "no" may overlap with NA in these instances.
folder	Folder name where the picture is stored (for internal use only)	Character
filename	Filename of the picture (for internal use only)	Character
country	Country where the data were collected	Brazil; Costa-Rica; Ecuador

B.1 Table 3. Site information

Variable identity	Variable definition	Unit/Levels of measurement
site	Site name, additional information are on file Site.txt	Character
midpoint_longitude	Longitude at the midpoint of the transect	WGS 84, EPSG:4326
midpoint_latitude	Latitude at the midpoint of the transect	WGS 84, EPSG:4326
extremity1_longitude	Longitude at one end of the transect	WGS 84, EPSG:4326
extremity1_latitude	Latitude at one end of the transect	WGS 84, EPSG:4326
extremity2_longitude	Longitude at the other end of the transect	WGS 84, EPSG:4326
extremity2_latitude	Latitude at the other end of the transect	WGS 84, EPSG:4326
min_transect_elev	Minimum elevation above sea level recorded along the transect. Calculated using the GPS track and the elevation raster at resolution 9.6 x 9.6 m from Terrain Tiles (accessed on 20 of June 2024 from	Meters above sea level

Variable identity	Variable definition	Unit/Levels of measurement
	https://registry.opendata.aws/terrain-tiles)	
max_transect_elev	Maximum elevation above sea level recorded along the transect. Calculated using the GPS track and the elevation raster at resolution 9.6 x 9.6 m from Terrain Tiles (accessed on 20 of June 2024 from https://registry.opendata.aws/terrain-tiles)	Meters above sea level
country	Country where the site is located	Brazil; Costa-Rica; Ecuador
size_of_reserve_ha	Size of the reserve where the site is located	Hectares (ha)
habitat	Type of habitat	forest; deforested
Description of forest	Type of forest	In Brazil: Old growth secondary; Intermediate/Old growth secondary; Intermediate. In Costa Rica: Old growth secondary; Secondary. In Ecuador: Primary; Cattle farm; Mixed primary and secondary; Old growth secondary.

B.1 Table 4. Transect (floral abundance)

Variable identity	Variable definition	Unit/Levels of measurement
date	Date of the flower census	YYYY-MM-DD
site	Site name, additional information are on file Site.txt	Character
flower_height	The height of each flower from the ground	Meters
count_method	Method for counting flowers	"DC" stands for Direct Count; "ES" stands for Estimation. NA means missing information

Variable identity	Variable definition	Unit/Levels of measurement
flowerAbundance	Total number of flowers for each plant	Number of open flowers counted on a given plant, though in some cases plants were in clumps and it was not possible to distinguish individual plants.
plant_scientificName	Full plant species scientific name according to the GBIF backbone taxonomy, with authorship and date information	Character
plant_usageKey	The GBIF taxonKey at the lowest taxonomic level, which is a unique identifier assigned by GBIF to a taxon in its backbone taxonomy.	Numeric
plant_species	Latin name of the plant species according to the GBIF taxonomy. If plant was not identified at species level, an ID of the unknown species is written, such as "Bromelia sp1"	Character
plant_genus	Plant taxonomy according to GBIF	Character
plant_family		Character
plant_order		Character
campaignID	Identifier linking flower census records to camera campaigns; censuses within ±5 days of a campaign interval receive the corresponding campaignID.	Character (site name + sequential number)
country	Country where the data were collected	Brazil; Costa-Rica; Ecuador

B.1 Table 5. Plant traits (flower traits)

Column name	Description	Unit/Levels
scientificName	Full plant species scientific name according to the GBIF backbone taxonomy, with authorship and date information	Character
usageKey	The GBIF taxonKey at the lowest	Numeric

Column name	Description	Unit/Levels
	taxonomic level, which is a unique identifier assigned by GBIF to a taxon in its backbone taxonomy.	
plant_species	Latin name of the plant species according to the GBIF taxonomy. If plant was not identified at species level, an ID of the unknown species was written, such as “Bromelia sp1”	Character
plant_genus	Plant taxonomy according to the GBIF backbone taxonomy	Character
plant_family		Character
plant_order		Character
taxonomic_level	Level of taxonomy associated with identification	Character
floralSymmetry	Corolla symmetry and whether it can be divided into two or more identical or mirror-image parts	actinomorphic; asymmetric; zygomorphic
flower_type	Type of flower	bilabiate; campanulate; funnelform; hypocrateriform; ligulate; tubular
flowerShape	Functional characterization of the flower or inflorescence shape	Bell; Brush; Dish; Flag; Gullet; Spurred; Tube
anther_position	Orientation of the stamens with respect to the flower main axis	dorsal; radial; semiradial; ventral
flower_position	Emergence of the flower on the stem	axillar; caulinar; terminal
petals_fusion	Petals fused or not fused	dialipetalous; gamopetalous
antherDehiscenceType	Physical mechanism of anther dehiscence and release of pollen grains	longitudinal; pore; transverse; rimose; valvate
coloredFlowerStructure	Organ involved in attracting the pollinator (corolla, bracts, leaves) was determined through the color palette	bract; flower; leaf; flower, bract; flower, leaf; flower, leaf, bract
tube_length	Length of the floral tube corresponds to the distance, in a straight line, from the base of the corolla to the corolla opening, without taking into account curvatures or protuberances	cm

Column name	Description	Unit/Levels
stamen_length	Measurement of the length of the stamen from the base of the calyx or hypanthium to the apical part without including the thecae, regardless of whether or not it arises from the base of the corolla or calyx.	cm
styleLengthInMilimiters	Length of style from base to the tip of the style	mm
corolla_opening_lateral	Width of the corolla in its lateral plane, at the opening of the floral tube, in species with zygomorphic flowers.	cm; or text “open_flower” for flowers with no tubular-like opening
corolla_opening_dorsal	Width of the corolla in its dorsal plane, at the opening of the floral tube	cm; or text “open_flower” for flowers with no tubular-like opening
corolla_curvature_central	Corolla curvature measured at the center of the flower as k=1/R. Curvature k of the fitted circumference, measured as 1 divided by the radius	cm-1
sexualSystem	Gender distribution in the flower or plant	cosexual; dioecious; monoecious
distyly_occurrence	Presence of floral polymorphism comprising two different floral phenotypes, long- and short-styled flowers	yes; no
sexual_maturation	Timing of maturation of male and female reproductive organs in cosexual flowers	homogamy; protandry
flowerOrientation	Position of the flowers with respect to the Cartesian plane	erect; horizontal; pendulous; erect_horizontal; pendulous_horizontal; various
resupination	Orientation of zygomorphic flowers during development resulting in a twist upside down of petals	yes; no
site	Site identity	Categorical
indiv_ID	Plant individual identity number	Integer
flower_ID	Flower identity number	Integer
country	Country identity	Brazil; Costa-Rica; Ecuador

B.1 Table 6. Herbarium records

Column name	Description	Unit/Levels
language	A language of the resource	Character; es, pt
collectionID	The identifier for the collection	Character; QCA, UPCB, USJ
catalogNumber	Unique identifier of the sample in the herbarium catalog	Numeric
usageKey	The GBIF taxonKey at the lowest taxonomic level, which is a unique identifier assigned by GBIF to a taxon in its backbone taxonomy	Numeric
scientificName	Name used when depositing the specimen at the herbarium	Character
acceptedNameUsage	Species full name used in the other EPHI spreadsheets, with authorship	Character
kingdom	Plant taxonomy according to the GBIF backbone taxonomy	Character
phylum		Character
order		Character
class		Character
family		Character
genus		Character
specificEpithet		Character
taxonRank	Lowest rank to which plant was identified	Character; Species, Genus, Kingdom, Variety
scientificNameAuthorship	Authorship of the scientific name	Character
identificationQualifier	A controlled value to express doubts about the ID	Character; cf., aff., sp. nov.
basisOfRecord	Nature of the data record	Character; PreservedSpecimen
institutionCode	Acronym of the institution	Character; QCA, USJ, UFPR
recordedBy	Individual who recorded the specimen	Character
identifiedBy	Individual who identifid the specimen	Character
eventDate	The date and time the specimen was collected	DateTime; YYYY-MM-DD HH:MM:SS
Location	Location where the specimen was collected	Character

site	Identifier of the location where the specimen was collected	Character; e.g., NIMB, LasGralarias
stateProvince	Provice where collection was made	Character
municipality	Manicipality where collection was made	Character
geodeticDatum	Spatial reference system used for coordinates	WGS 84, EPSG:4326
decimalLatitude	Latitude of the sampling location in decimal degrees	Numeric; decimal degrees
decimalLongitude	Longitude of the sampling location in decimal degrees	Numeric; decimal degrees
minimumElevationInMeters	Minimum elevation of the sampling	Numeric; Meters above sea level
maximumElevationInMeters	Maximum elevation of the sampling	Numeric; Meters above sea level
countryCode	Standard code for the country in which the specimen was collected	Character; EC, BR, CR
country	Country where the specimen was collected	Character; Brazil; Costa Rica; Ecuador

R scripts

Merging_atlantic_pollination.R: R script to merge our dataset with the Atlantic Pollination dataset (Iamara-Nogueira et al. 2022). This script standardizes plant and animal taxonomy according to the Global Biodiversity Information Facility (GBIF), ensuring consistency across both datasets. Additionally, it aligns categorical flower shape traits, calculates the average and standard deviation of tube length—data available in the Atlantic Pollination dataset—for our records, and adjusts column names so that shared information, such as species names, sampling duration, and feeding behavior (legitimate visits or nectar robbing), are stored under the same columns in the merged dataset. Note that users can use the transect data we provide to derive the first month of flowering ("flowering start month") and the number of months with flowers ("flowering period month"), which are available in the

Atlantic Pollination dataset. We do not provide these calculations in the R script, as they require a series of user-defined decisions (e.g., handling data from two years of sampling). The user can choose between generating a data.frame with all columns from both datasets, in which case NA's will be added whenever an information is not available in one of the datasets, or generating a data.frame that only includes the columns that are in common between both datasets.

DarwinCorePPI.R: code to transform the original data structure and vocabulary into a Darwin Core + Plant–Pollinator Interactions compliant version (Salim et al. 2022). Only needed if the user wants to regenerate or adapt the transformation process. Otherwise, we provide the already formatted data in DarwinCorePPI_compliant.zip.

C. **Data anomalies**: Missing data are denoted by NA.

Class V. Supplemental descriptors

A. Data acquisition:

1. Data forms or acquisition methods: We used standardized hard copy data forms for data acquisition across all countries. On these forms all information was taken in a standardized way (i.e., same coding and recording methods). We had two data sheets for data collection in the field: 1) transect data sheet where we entered data on flora abundance from the transect (Figure 13a) and 2) camera data sheet where we noted information about the plant species filmed (Figure 13b).

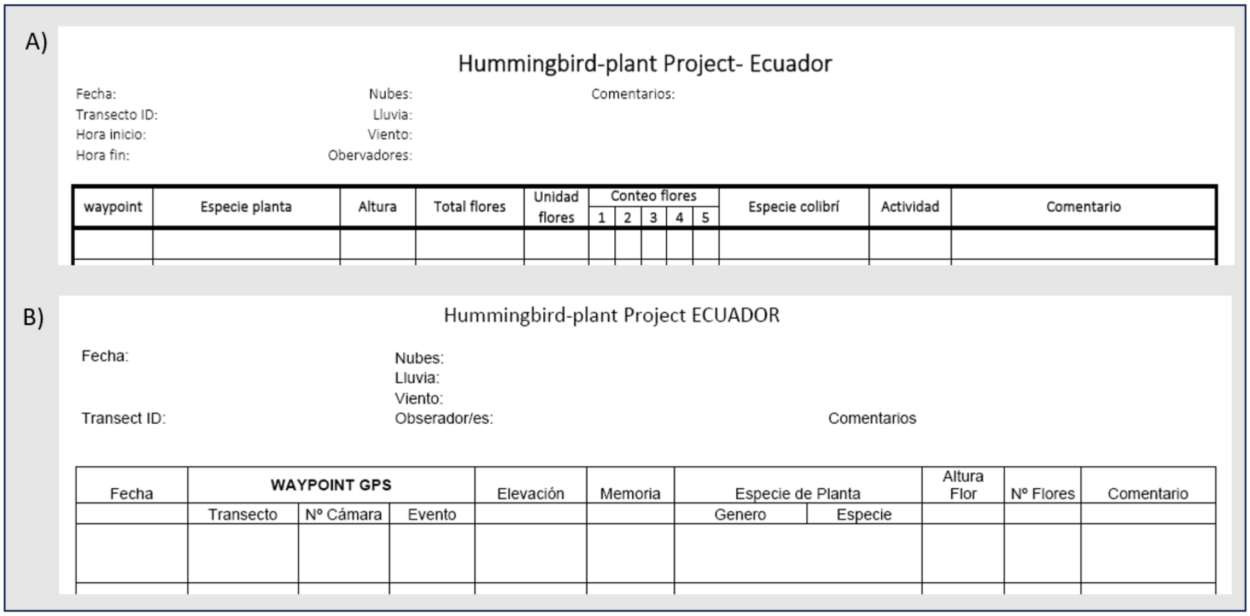

A)

Hummingbird-plant Project- Ecuador

Fecha:
Transecto ID:
Hora inicio:
Hora fin:
Nubes:
Lluvia:
Viento:
Obervadores:
Comentarios:

waypoint	Especie planta	Altura	Total flores	Unidad flores	Conteo flores					Especie colibrí	Actividad	Comentario
					1	2	3	4	5			

B)

Hummingbird-plant Project ECUADOR

Fecha:
Nubes:
Lluvia:
Viento:
Transect ID:
Obserador/es:
Comentarios

Fecha	WAYPOINT GPS			Elevación	Memoria	Especie de Planta		Altura Flor	Nº Flores	Comentario
	Transecto	Nº Cámara	Evento			Genero	Especie			

Figure 13: Example data sheets for field data entry for a) flower data take on transects and b) camera placement information.

2. Data entry: The data from the two field data sheets were entered by the same person who collected the data usually within one week of data collection (referred to as digital sheets). All data were checked for errors (see below). Additionally, we had a third data sheet where we identified interactions from the cameras.

3. Data entry verification procedures: We implemented a two-step data verification process during and after data collection. During the data collection phase, data were continuously verified using a Shiny app written in R language developed for internal use. This tool was designed to identify and report inconsistencies in real time, specifically flagging the items listed below. The person who inputted the data then checked the data sheet or camera frames as needed and corrected the data. The tool was then rerun on the data. This process was repeated until the tool reported no errors. Items flagged by the tool:

- *Plant names*: The report indicated the missing plant species, the digital sheet where it was missing (transect, cameras, or interactions), and the site. A plant was considered missing if it was misspelled (typos, capitalization, spaces) or if its name did not match the accepted name in Tropicos.org (eventually replaced by the GBIF taxonomy for the final version of the data).
- *Hummingbird names*: The report was similar to the one for plants but flagged missing hummingbird species, either because they were misspelled or did not match the accepted name in BirdLife, later replaced by The American Ornithological Society's (AOS) Checklist and ultimately GBIF.
- *Missing waypoints*: Similar to the previous reports, this flagged missing waypoints in different sheets and sites. The names assigned to waypoints needed to match across the data sheets, interaction sheets, and the .gpx file from the GPS used to record the data in the field.

After completing the data collection, a second verification step focused on stardardizing the dataset across all countries involved. This step emphasized the following:

- *Taxonomic standardization*: We standardized plant and hummingbird species names by ensuring consistent formatting (e.g., spacing, no special character, checking uppercase and lowercase letters). Next, we performed taxonomic homogenization based on GBIF taxonomy using the R package *gbif* (Chamberlain and Boettiger 2017, Chamberlain et al. 2024). In this case the tool flagged any mismatches in taxonomy that were then manually correct as described above.

- *Categorical variable standardization*: We standardized the levels of all categorical variables. For example, hummingbird sex data such as "mj," "Male," "male," and "young male" were all unified as "m".
- *Date and time formatting*: All date and time fields were formatted using R's standard system functions.
- *Date and time consistency check*: We ensured that the dates and times of all recorded interactions for a given plant individual were within the recording intervals of that camera. This was done by cross-checking the interaction dates and times in the interaction file with the camera's operational times in the camera file.
- *Duplicate waypoint*: Duplicate waypoints (ID's) were identified and resolved by verifying associated interactions based on date and time or manually reviewing the original data.
- *Outlier detection*: Outliers in continuous data, such as floral abundance and trait data, were flagged for verification against the raw data.
- *Missing data*: Missing data across all cells were flagged.

Any inconsistencies were cross-referenced with raw paper files and interaction pictures.

B. Quality assurance/quality control procedures:

Before data collection: In each country data takers practiced together to ensure consistency of data acquisition and recording. Workshops were run where each data taker walked a single transect and took all data. In particular, all estimated flowers based extrapolation as described above. After this, the data sheets were compared to determine the extent to which the same flowers had been identified and counted. Finally, the data takers walked the transect together

pointing out missed flowers to each other and practiced the estimation together when these were more than ~10% different. We repeated this exercise as necessary until recorded data was similar across data recorders. Additionally, all those who collected data were trained in the use and placement of cameras, use of GPS, use of the field sheet, data entry, video analysis, and identification of interactions. This was done with exercises in the field and office following established and detailed protocol instructions.

After data collection: See "Data entry verification procedures" above

Related materials: None

Computer programs and data-processing algorithms: Deep meerkat was used to identify frames with hummingbirds as described above (Weinstein 2015). All data were entered into excel. A shiny app in R language was developed for internal use, to flag any inconsistencies when digitizing the data. The data were further checked and cleaned using a series of R scripts available in the supplementary material.

C. Archiving:

We conserved the physical completed data forms at the original institutions which were responsible for data collection: Aves y Conservación in Ecuador, UNED in Costa Rican and UFPR in Brazil. The virtual data are on Dropbox folders, one for each country. These are backed up on a server at the Swiss Federal Institute for Forest, Snow and Landscape Research (WSL).

D. Publications and results:

Parts of the dataset have been used in the following peer-reviewed or pre-print publications (Barreto et al. 2025, Duchenne et al. 2022, 2023, 2025, Guevara et al. 2023b, 2023a, de Oliveira et al. 2025, Sánchez-Martín et al. 2025a, 2025b).

- Barreto, E., Duchenne, F., Beck, H., Bello, C., Bobato, R., Brenes, E., Bôlla, D., Büttner, N., Caron, A.P., Castro, J.A., Chaves-Elizondo, N., Gavilanes, M.J., Guevara, E.A., Kaehler, M., Machado-de-Souza, T., Machnicki-Reis, M., Marcayata-Fajardo, A.S., de Menezes, C.G., Nieto, A., de Oliveira, R., de Oliveira, R.A.C., Restrepo-González, A., Richter, F., Rojas, B.G., Romanowski, L.L., Sánchez-Martín, R., da Silva, R.C., de Souza, W.L., Veluza, D.S., Weinstein, B., Wüest, R., Zanata, T.B., Zuniga, K., Santander, T., Maglianesi, M.A., Varassin, I.G. and Graham, C.H. (2025), Hummingbird flower visitation rates vary with species traits, floral abundance and phenology across bioregions. Oikos, 2025: e11354.
- Duchenne, F., R. O. Wüest, and C. H. Graham. 2022. Seasonal structure of interactions enhances multidimensional stability of mutualistic networks. Proceedings of the Royal Society B: Biological Sciences 289:20220064.
- Duchenne, F., S. Aubert, E. Barreto, E. Brenes, M. A. Maglianesi, T. Santander, E. A. Guevara, and C. H. Graham. 2023. When cheating turns into a stabilizing mechanism of plant–pollinator communities. PLOS Biology 21:e3002434.
- Duchenne, F., E. Barreto, E. A. Guevara, H. Beck, C. Bello, R. Bobato, D. Bôlla, E. Brenes, N. Büttner, A. P. Caron, N. Chaves-Elizondo, M. J. Gavilanes, A. Restrepo-González, J. A. Castro, M. Kaehler, T. Machado-de-Souza, M. Machnicki-Reis, A. S. F. Marcayata, C. G. de Menezes, A. Nieto, R. de Oliveira, R. A. C. de Oliveira, F. Richter, B. G. Rojas, L. L. Romanowski, W. L. de Souza, D. S. Veluza, B. Weinstein, R. O.

Wüest, T. B. Zanata, K. Zuniga, M. A. Maglianesi, T. Santander, I. G. Varassin, and C. H. Graham. 2025. A Probabilistic View of Forbidden Links: Their Prevalence and Their Consequences for the Robustness of Plant–Hummingbird Communities. Ecology Letters 28:e70073.

- Guevara, E. A., C. Bello, C. Poveda, I. R. McFadden, M. Schleuning, L. Pellissier, and C. H. Graham. 2023a. Hummingbird community structure and nectar resources modulate the response of interspecific competition to forest conversion. Oecologia 201:761–770.
- Guevara, E. A., D. M. Dehling1, C. Bello, M. Schleuning, L. Pellissier, T. S. G, F. Tobar, and C. H. Graham. 2023b, January 31. Asymmetric effect of deforestation on the functional roles of interacting plants and hummingbirds.
- de Oliveira, R., E. Barreto, T. B. Zanata, F. Tobar, T. Santander, M. J. Gavilanes, C. H. Graham, and I. G. Varassin. 2025. Niche packing, but not niche expansion, explains the co-occurrence of hummingbirds-visited plants. Ecography 2025:e07440:1–12.
- Sánchez-Martín, R., Barreto, E., Maxwell, M. F., Duchenne, F., Beck, H., Bobato, R., Brenes, E., Bôlla, D., Büttner, N., Caron, A. P., Castro Jiménez, A., Chaves-Elizondo, N., Gavilanes, M. J., Görlich, A. S., Guevara, E. A., Kaehler, M., Machado De Souza, T., Machnicki-Reis, M., Sebastián Marcayata-Fajardo, A., … Graham, C. H. (2025). Mechanisms influencing network topology in plant–hummingbird pollination networks. Proceedings B, 292(2059).
- Sánchez-Martín, R., Barreto, E., Duchenne, F., Varassin, I. G., Maglianesi, M., Tinoco, B., & Graham, C. H. (2025). Functional generalism in plant–hummingbird interactions: Causes and consequences from a plant perspective. Functional Ecology, 39, 3339–3350.

And the following doctoral and master thesis:

- Esteban A. Guevara. 2022. The effects of land-use change on species interactions in the Tropical Andes. Doctoral dissertation ETH Zürich.
- Bryan Gastón Rojas González. 2022. Floral density-dependent effects on plant-hummingbird interactions along an elevation gradient. Master thesis in Ecology and Conservation. Universidade Federal do Paraná.
- Danila Syriani Veluza. 2023 Balanço entre taxa de visitação de beija-flores e deposição de pólen em plantas raras e abundantes. 2023. Master thesis in Botany. Universidade Federal do Paraná.
- Heloisa Ribeiro. 2025. Floral trait similarity in co-flowering modules in a hummingbird pollination system. Master thesis in Ecology and Conservation. Universidade Federal do Paraná.

E. **History of data set usage**: Parts of the dataset have been used in the above publications.

Acknowledgments

In Ecuador, the project was led by Aves y Conservación / BirdLife in Ecuador, and data collection was possible thanks the local assistance of Wilson Hipo, Rolando Hipo, Silvio Calderón, Roberto Pailacho, Wilfrido Vaca, Christian Montalvo, Leo Montalvo, Noé Morales, Edison Tapia, Segundo Imba, Gera Obando, Daniel Ponce, Diana Salvador, Michael Carlosama, Sergio Basantes, Antonio Páez, and Adela Espinoza. We are also grateful to Santa Lucía, Maquipucuna Foundation, Un Poco del Chocó, Mashpi Lodge, Las Gralarias Foundation, Sachatamia Lodge, Jocotoco Foundation, Verdecocha, Casa Rumisitana, Puyucunapi, Amagusa,

and the Alaspungo community. We also thank the National Herbarium and the QCA Herbarium (Ecuador) for access to their collections and the Ministry of Environment in Ecuador for providing the research permit to conduct fieldwork.

In Costa Rica, we thank José Alejandro Castro Jiménez, Fabian Monge Badilla, Greivin Serrano Salazar, Samael Padilla, Yandry Hernadez Barboza, Karen Garro Alvarado, Silvia Cascante Chinchilla, Pablo Gutiérrez Campos, Margarita Valverde Quesada, Greilyn Fallas Rodriguez, Lizet Hidalgo Picado, Xiomara Castro Arroyo, Gabriel Zuniga Morales, Guisselle Arce Soto. We also thank the owners and administrators of the study sites, Los Nimbulos, Los Amigos del Bosque, Villa Mills Experimental Station (ACC), Villa San Miguel, Finca Boquete (Sibu), Bosque del Tolomuco, Centro Biológico Las Quebradas (FUDEBIOL), Las Nubes (York University), Sendero Los Gigantes, Refugio de Aves Los Cusingos (CCT), Hotel Rio Magnolia, Longo Mai, and La Georgina. We are grateful to the Sistema Nacional de Áreas de Conservación (SINAC) for facilitating research permits to access wild areas.

In Brazil, we thank Parque Estadual Serra do Mar - Núcleo Picinguaba e Núcleo Cunha - SP, RPPN Instituto Alto Montana da Serra Fina - MG, Fazenda Bananal - Paraty - RJ, Parque Nacional do Itatiaia - RJ, Instituto Chico Mendes de Conservação da Biodiversidade (ICMBIO), Instituto de Pesquisas Ambientais (IPA), and Sistema Nacional de Gestão do Patrimônio Genético e do Conhecimento Tradicional Associado (SISGEN) for study and research permits.

Literature Cited

Abrahamczyk, S., and M. Kessler. 2015. Morphological and behavioural adaptations to feed on nectar: how feeding ecology determines the diversity and composition of hummingbird assemblages. Journal of Ornithology 156:333–347.

Barreto, E., Duchenne, F., Beck, H., Bello, C., Bobato, R., Brenes, E., Bôlla, D., Büttner, N., Caron, A.P., Castro, J.A., Chaves-Elizondo, N., Gavilanes, M.J., Guevara, E.A., Kaehler, M., Machado-de-Souza, T., Machnicki-Reis, M., Marcayata-Fajardo, A.S., de Menezes, C.G., Nieto, A., de Oliveira, R., de Oliveira, R.A.C., Restrepo-González, A., Richter, F., Rojas, B.G., Romanowski, L.L., Sánchez-Martín, R., da Silva, R.C., de Souza, W.L., Veluza, D.S., Weinstein, B., Wüest, R., Zanata, T.B., Zuniga, K., Santander, T., Maglianesi, M.A., Varassin, I.G. and Graham, C.H. (2025), Hummingbird flower visitation rates vary with species traits, floral abundance and phenology across bioregions. Oikos, 2025: e11354.

Bascompte, J., and P. Jordano. 2013. Mutualistic Networks. 1st edition. Princeton University Press, Princeton, Nova Jersey, EUA.

Bleiweiss, R. 1998. Tempo and mode of hummingbird evolution. Biological Journal of the Linnean Society 65:63–76.

Carranza-Quiceno, J. A., J. H. Castaño, S. B. Muriel-Ruiz, P. K. M. Mendoça, and I. Armbrecht. 2024. Forest loss and habitat changes reduce hummingbird functional diversity and specialization of their interactions with plants in the tropical Andes. Global Ecology and Conservation:e03062.

Chakraborty, A. 2021. Mountains as vulnerable places: a global synthesis of changing mountain systems in the Anthropocene. GeoJournal 86:585–604.

Chamberlain, S., V. Barve, D. Mcglinn, D. Oldoni, P. Desmet, L. Geffert, and K. Ram. 2024. rgbif: Interface to the Global Biodiversity Information Facility API. R.

Chamberlain, S., and C. Boettiger. 2017. R Python, and Ruby clients for GBIF species occurrence data. PeerJ PrePrints.

Clark, J. L., M. M. Funke, A. M. Duffy, and J. F. Smith. 2012. Phylogeny of a Neotropical Clade in the Gesneriaceae: More Tales of Convergent Evolution. International Journal of Plant Sciences 173:894–916.

Clark, J. L., and F. Tobar. 2021. *Glossoloma wiehleri* (Gesneriaceae), a new species from the northwestern Andes of Ecuador. PhytoKeys 186:1–9.

Clark, J. L., F. Tobar, L. Clavijo, M. Perret, and C. H. Graham. 2021. Three new species of *Columnea* (Gesneriaceae) from the western Andean slopes of Ecuador and Colombia. PhytoKeys 182:67–82.

Colwell, R. K., T. F. Rangel, K. Fučíková, D. Sustaita, G. Yanega M., and A. Rico-Guevara. 2023. Repeated Evolution of Unorthodox Feeding Styles Drives a Negative Correlation between Foot Size and Bill Length in Hummingbirds. American Naturalist 202:699–719.

Dalsgaard, B., P. K. Maruyama, J. Sonne, K. Hansen, T. B. Zanata, S. Abrahamczyk, R. Alarcón, A. C. Araujo, F. P. Araújo, S. Buzato, E. Chávez-González, A. G. Coelho, P. A. Cotton, R. Díaz-Valenzuela, M. F. Dufke, P. L. Enríquez, M. Martins Dias Filho, E. Fischer, G. Kohler, C. Lara, F. M. G. Las-Casas, L. Rosero Lasprilla, A. O. Machado, C. G. Machado, M. A. Maglianesi, T. S. Malucelli, O. H. Marín-Gómez, V. Martínez-García, S. Mendes de Azevedo-Júnior, E. N. da Silva Neto, P. E. Oliveira, J. F. Ornelas, R. Ortiz-Pulido, R. Partida-Lara, B. I. Patiño-González, S. Najara de Pinho Queiroz, M. B. Ramírez-Burbano, A. Rech, M. A. Rocca, L. C. Rodrigues, A. M. Rui, I. Sazima, M. Sazima, B. I. Simmons, B. A. Tinoco, I. G. Varassin, M. F. Vasconcelos, J. Vizentin-Bugoni, S. Watts, J. D. Kennedy, C. Rahbek, M. Schleuning, and A. M. Martín González. 2021. The influence of biogeographical and evolutionary histories on morphological trait-matching and resource specialization in mutualistic hummingbird–plant networks.

Functional Ecology 35:1120–1133.

Dellinger, A. S., A. M. Hamilton, C. A. Wessinger, and S. Smith. 2023. Opposing patterns of altitude-driven pollinator turnover in the tropical and temperate Americas. The American naturalist 202:152–165.

Duchenne, F., S. Aubert, E. Barreto, E. Brenes, M. A. Maglianesi, T. Santander, E. A. Guevara, and C. H. Graham. 2023. When cheating turns into a stabilizing mechanism of plant–pollinator communities. PLOS Biology 21:e3002434.

Duchenne, F., E. Barreto, E. A. Guevara, H. Beck, C. Bello, R. Bobato, D. Bôlla, E. Brenes, N. Büttner, A. P. Caron, N. Chaves-Elizondo, M. J. Gavilanes, A. Restrepo-González, J. A. Castro, M. Kaehler, T. Machado-de-Souza, M. Machnicki-Reis, A. S. F. Marcayata, C. G. de Menezes, A. Nieto, R. de Oliveira, R. A. C. de Oliveira, F. Richter, B. G. Rojas, L. L. Romanowski, W. L. de Souza, D. S. Veluza, B. Weinstein, R. O. Wüest, T. B. Zanata, K. Zuniga, M. A. Maglianesi, T. Santander, I. G. Varassin, and C. H. Graham. 2025. A Probabilistic View of Forbidden Links: Their Prevalence and Their Consequences for the Robustness of Plant–Hummingbird Communities. Ecology Letters 28:e70073.

Duchenne, F., R. O. Wüest, and C. H. Graham. 2022. Seasonal structure of interactions enhances multidimensional stability of mutualistic networks. Proceedings of the Royal Society B: Biological Sciences 289:20220064.

Faegri, K., and L. van der Pijl. 1979. Principles of pollination ecology. Elsevier.

Feinsinger, P., and R. K. Colwell. 1978. Community Organization Among Neotropical Nectar-Feeding Birds. American Zoologist 18:779–795.

Feinsinger, P., R. K. Colwell, J. Terborgh, and S. B. Chaplin. 1979. Elevation and the Morphology, Flight Energetics, and Foraging Ecology of Tropical Hummingbirds. The

American Naturalist 113:481–497.

GBIF Secretariat. 2025 . GBIF Backbone Taxonomy. accessed via GBIF.org on 08 of September 2025.

Gill, F., D. Donsker, and P. Rasmussen. 2024. IOC World Bird List.

González, M. P., E. Bonaccorso, and M. Papeş. 2015. Applications of geographic information systems and remote sensing techniques to conservation of amphibians in northwestern Ecuador. Global Ecology and Conservation 3:562–574.

Graham, C. H., J. L. Parra, C. Rahbek, and J. A. McGuire. 2009. Phylogenetic structure in tropical hummingbird communities. Proceedings of the National Academy of Sciences 106:19673–19678.

Grant, K. A., and V. Grant. 1968. Hummingbirds and their flowers. Columbia University Press, New York.

Guevara, E. A., C. Bello, C. Poveda, I. R. McFadden, M. Schleuning, L. Pellissier, and C. H. Graham. 2023a. Hummingbird community structure and nectar resources modulate the response of interspecific competition to forest conversion. Oecologia 201:761–770.

Guevara, E. A., D. M. Dehling, C. Bello, M. Schleuning, L. Pellissier, T. S. G, F. Tobar, and C. H. Graham. 2023b. Asymmetric effect of deforestation on the functional roles of interacting plants and hummingbirds. Research Square.

Hadley, A. S., S. J. K. Frey, W. D. Robinson, and M. G. Betts. 2018. Forest fragmentation and loss reduce richness, availability, and specialization in tropical hummingbird communities. Biotropica 50:74–83.

Holdridge, L. R. 1967. Life zone ecology. Tropical Science Center, San José, Costa Rica.

Humboldt, A. von, and A. Bonpland. 2013. Essay on the Geography of Plants. Page (S. T. J. T.

by S. Romanowski, Tran.). University of Chicago Press, Chicago, IL.

Iamara-Nogueira, J., N. Targhetta, G. Allain, A. Gambarini, A. R. Pinto, A. M. Rui, A. C. Araújo, A. Lopes, B. Pereira-Silva, B. B. de Camargo, C. G. Machado, C. Missagia, C. Scultori, D. Boscolo, E. Fischer, E. S. Araújo-Oliveira, H. Gava, H. F. Paulino-Neto, I. C. Machado, I. G. Varassin, I. Sazima, J. Vizentin-Bugoni, J. L. S. Silva, J. de Oliveira Ferreira, J. Narita, J. S. dos Santos, K. Agostini, L. Freitas, L. E. Lopes, L. J. Carvalho-Leite, M. Tabarelli, M. A. Rocca, M. L. Malanotte, M. A. S. Alves, M. B. F. Canela, M. R. Darrigo, M. M. Moreira, M. Wolowski, M. Sazima, M. Galetti, M. C. Ribeiro, M. Groppo, M. Kaehler, M. dos A. Batista, O. C. Neto, P. A. Ferreira, P. J. Bergamo, P. K. Maruyama, R. O. Bueno, R. L. B. Leal, R. R. Faria, S. Bazarian, T. Malucelli, and S. Buzato. 2022. ATLANTIC POLLINATION: a data set of flowers and interaction with nectar-feeding vertebrates from the Atlantic Forest. Ecology 103:e03595.

Instituto Nacional de Meteorologia. 2015. Banco de dados meteorológicos.

Janzen, D. H. 1967. Why Mountain Passes are Higher in the Tropics. The American Naturalist 101:233–249.

Kattan, G. H., P. Franco, V. Rojas, and G. Morales. 2004. Biological diversification in a complex region: a spatial analysis of faunistic diversity and biogeography of the Andes of Colombia. Journal of Biogeography 31:1829–1839.

de Lima, R. F., L. E. de Oliveira Aparecido, J. A. Lorençone, P. A. Lorençone, G. B. Torsoni, J. R. da Silva Cabral Moraes, and K. C. de Meneses. 2022. Assessing life zone changes under climate change scenarios in Brazil. Theoretical and Applied Climatology 149:1687–1703.

Maruyama, P. K., C. Bonizário, A. P. Marcon, G. D’Angelo, M. M. da Silva, E. N. da Silva

Neto, P. E. Oliveira, I. Sazima, M. Sazima, J. Vizentin-Bugoni, L. dos Anjos, A. M. Rui, and O. Marçal Júnior. 2019. Plant-hummingbird interaction networks in urban areas: Generalization and the importance of trees with specialized flowers as a nectar resource for pollinator conservation. Biological Conservation 230:187–194.

Maruyama, P. K., G. M. Oliveira, C. Ferreira, B. Dalsgaard, and P. E. Oliveira. 2013. Pollination syndromes ignored: importance of non-ornithophilous flowers to Neotropical savanna hummingbirds. Naturwissenschaften 100:1061–1068.

McGuire, J. A., C. C. Witt, J. V. Remsen, A. Corl, D. L. Rabosky, D. L. Altshuler, and R. Dudley. 2014. Molecular Phylogenetics and the Diversification of Hummingbirds. Current Biology 24:910–916.

Ministerio del Ambiente del Ecuador. 2013. Sistema de Clasificación de los Ecosistemas del Ecuador Continental. Subsecretaría de Patrimonio Natural, Quito, Ecuador.

Morrison, B. M. L., and C. D. Mendenhall. 2020. Hummingbird–Plant Interactions Are More Specialized in Forest Compared to Coffee Plantations. Diversity 12:126.

Myers, N., R. A. Mittermeier, C. G. Mittermeier, G. A. B. da Fonseca, and J. Kent. 2000. Biodiversity hotspots for conservation priorities. Nature 403:853–858.

de Oliveira, R., E. Barreto, T. B. Zanata, F. Tobar, T. Santander, M. J. Gavilanes, C. H. Graham, and I. G. Varassin. 2025. Niche packing, but not niche expansion, explains the co-occurrence of hummingbirds-visited plants. Ecography 2025:e07440:1–12.

Ornelas, J. F., M. Ordano, A. J. De-Nova, M. E. Quintero, and T. Garland. 2007. Phylogenetic analysis of interspecific variation in nectar of hummingbird-visited plants: The evolution of nectar. Journal of Evolutionary Biology 20:1904–1917.

Pellissier, L., C. Albouy, J. Bascompte, N. Farwig, C. Graham, M. Loreau, M. A. Maglianesi, C.

J. Melián, C. Pitteloud, T. Roslin, R. Rohr, S. Saavedra, W. Thuiller, G. Woodward, N. E. Zimmermann, and D. Gravel. 2018. Comparing species interaction networks along environmental gradients: Networks along environmental gradients. Biological Reviews 93:785–800.

Pyke, G. H., and N. M. Waser. 1981. The Production of Dilute Nectars by Hummingbird and Honeyeater Flowers. Biotropica 13:260–270.

Rahbek, C., M. K. Borregaard, R. K. Colwell, B. Dalsgaard, B. G. Holt, N. Morueta-Holme, D. Nogues-Bravo, R. J. Whittaker, and J. Fjeldså. 2019. Humboldt's enigma: What causes global patterns of mountain biodiversity? Science 365:1108–1113.

Rodríguez, F., A. Castro, F. Marín, G. Roldán, and F. V. Moya. 2019. Typical meteorological year based on the precipitation of Nanegalito and Pacto-Ecuador. Enfoque UTE 10:197–204.

Rodríguez-Flores, C. I., J. F. Ornelas, S. Wethington, and M. del C. Arizmendi. 2019. Are hummingbirds generalists or specialists? Using network analysis to explore the mechanisms influencing their interaction with nectar resources. PLOS ONE 14:e0211855.

Salim, J. A., Saraiva, A. M., Zermoglio, P. F., Agostini, K., Wolowski, M., Drucker, D. P., Soares, F. M., Bergamo, P. J., Varassin, I. G., Freitas, L., Maués, M. M., Rech, A. R., Veiga, A. K., Acosta, A. L., Araujo, A. C., Nogueira, A., Blochtein, B., Freitas, B. M., Albertini, B. C., Maia-Silva, C., Nunes, C. E. P., Pires, C. S. S., dos Santos, C. F., Queiroz, E. P., Cartolano, E. A., de Oliveira, F. F., Amorim, F. W., Fontúrbel, F. E., da Silva, G. V., Consolaro, H., Alves-dos-Santos, I., Machado, I. C., Silva, J. S., Aleixo, K. P., Carvalheiro, L. G., Rocca, M. A., Pinheiro, M., Hrncir, M., Streher, N. S., Ferreira, P. A., de Albuquerque, P. M. C., Maruyama, P. K., Borges, R. C., Giannini, T. C., Brito, V.

L. G. Data standardization of plant–pollinator interactions, GigaScience, Volume 11, 2022.

Sánchez-Martín, R., Barreto, E., Maxwell, M. F., Duchenne, F., Beck, H., Bobato, R., Brenes, E., Bôlla, D., Büttner, N., Caron, A. P., Castro Jiménez, A., Chaves-Elizondo, N., Gavilanes, M. J., Görlich, A. S., Guevara, E. A., Kaehler, M., Machado De Souza, T., Machnicki-Reis, M., Sebastián Marcayata-Fajardo, A., … Graham, C. H. (2025). Mechanisms influencing network topology in plant–hummingbird pollination networks. Proceedings B, 292(2059).

Sánchez-Martín, R., Barreto, E., Duchenne, F., Varassin, I. G., Maglianesi, M., Tinoco, B., & Graham, C. H. (2025). Functional generalism in plant–hummingbird interactions: Causes and consequences from a plant perspective. Functional Ecology, 39, 3339–3350.

Santander, T., I. Varassin, M. A. Maglianesi, S. Aubert, E. Barreto, H. Beck, C. Bello, R. Bobato, E. Brenes, D. Bôlla, et al. 2025. Plant-hummingbird interactions, floral abundance and floral traits for three altitudinal gradients in the Americas. Zenodo. https://doi.org/10.5281/zenodo.17092717

Serrano-Serrano, M. L., J. Rolland, J. L. Clark, N. Salamin, and M. Perret. 2017. Hummingbird pollination and the diversification of angiosperms: an old and successful association in Gesneriaceae. Proceedings of the Royal Society B: Biological Sciences 284:20162816.

Sonne, J., and C. Rahbek. 2024. Idiosyncratic patterns of local species richness and turnover define global biodiversity hotspots. Proceedings of the National Academy of Science 121:e2313106121.

Stiles, F. G. 2004. Phylogenetic constraints upon morphological and ecological adaptation in hummingbirds (Trochilidae): why are there no hermits in the Paramo? Ornitologia

Neotropical 15:191–198.

Stiles, F. G. 2008. Ecomorphology and phylogeny of hummingbirds: divergence and convergence in adaptations to high elevations. Ornitologia Neotropical 19:511–519.

Suarez, F. T., M. F. López, M. J. Gavilanes, M. F. Monteros, T. S. García, and C. H. Graham. 2021. Three new endemic species of *Lepanthes* (Orchidaceae, Pleurothallidinae) from the highlands of Ecuador. PhytoKeys 180:111–132.

Tinoco, B. A., V. E. Santillán, and C. H. Graham. 2018. Land use change has stronger effects on functional diversity than taxonomic diversity in tropical Andean hummingbirds. Ecology and Evolution 8:3478–3490.

Tobar, F., H. Beck, M. J. Gavilanes, T. G. Santander, Á. J. Pérez, D. Cevallos, E. Rea, and C. H. Graham. 2022a. Plants of Santa Lucía Reserve visited by hummingbirds. Rapid Color Guides, Field Museum.

Tobar, F., H. Beck, M. J. Gavilanes, T. G. Santander, Á. J. Pérez, E. Rea, D. Cevallos, and C. H. Graham. 2022b. Plants of Maquipucuna Reserve visited by hummingbirds. Rapid Color Guides, Field Museum.

Tobar, F., M. J. Gavilanes, C. Poveda, E. A. Guevara, T. G. Santander, A. Marcayata, Á. J. Pérez, D. Cevallos, E. Rea, B. G. Rojas, and C. H. Graham. 2022c. Plants of Alaspungo Reserve visited by hummingbirds. Rapid Color Guides, Field Museum.

Tobar, F., E. A. Guevara, T. G. Santander, C. Poveda, M. J. Gavilanes, Á. J. Pérez, B. G. Rojas, D. Cevallos, E. Rea, and C. H. Graham. 2022d. Plants of Puyucunapi Reserve visited by hummingbirds. Rapid Color Guides, Field Museum.

Tobar, F., E. Guevara, M. J. Gavilanes, C. Poveda, A. Marcayata, A. Nieto, Á. J. Pérez, B. G. Rojas, D. Cevallos, E. Rea, and C. H. Graham. 2022e. Plants of Sachatamia Reserve

visited by hummingbirds. Rapid Color Guides, Field Museum.

Tobar, F., E. Guevara, T. G. Santander, C. Poveda, M. J. Gavilanes, Á. J. Pérez, B. G. Rojas, D. Cevallos, E. Rea, and C. H. Graham. 2022f. Plants of Rumisitana visited by hummingbirds. Rapid Color Guides, Field Museum.

Tobar, F., A. Nieto, E. Guevara, M. J. Gavilanes, F. Richter, Á. J. Pérez, A. Marcayata, E. Rea, D. Cevallos, and C. H. Graham. 2020a. Plants of Las Gralarias Reserve visited by hummingbirds. Rapid Color Guides, Field Musem.

Tobar, F., A. Nieto, F. Richter, M. J. Gavilanes, T. G. Santander, A. Marcayata, Á. J. Pérez, D. Cevallos, E. Guevara, E. Rea, and C. H. Graham. 2021. Plants of Mashpi Reserve visited by hummingbirds. Rapid Color Guides, Field Museum.

Tobar, F., T. G. Santander, E. A. Guevara, Á. J. Pérez, E. Rea, D. Cevallos, W. Artega, C. Poveda, B. G. Rojas, and C. H. Graham. 2020b. Plants of Yanacocha and Verdecocha Reserve visited by hummingbirds. Rapid Color Guides, Field Museum.

Tobar, F., J. F. Smith, and J. L. Clark. 2022g. Two new pendulous epiphytic *Columnea* L. (Gesneriaceae) species from the Chocó forests of the Northern Andes. PhytoKeys 196:49–61.

Waser, N. M., P. J. CaraDonna, and M. V. Price. 2018. Atypical Flowers Can Be as Profitable as Typical Hummingbird Flowers. The American Naturalist 192:644–653.

Weinstein, B. G. 2015. MotionMeerkat: integrating motion video detection and ecological monitoring. Methods in Ecology and Evolution 6:357–362.

Weinstein, B. G., and C. H. Graham. 2017. Persistent bill and corolla matching despite shifting temporal resources in tropical hummingbird-plant interactions. Ecology Letters 20:326–335.
